# *ADORA2A* variation and adenosine A_1_ receptor availability in the human brain with a focus on anxiety-related brain regions: modulation by *ADORA1* variation

**DOI:** 10.1038/s41398-020-01085-w

**Published:** 2020-11-24

**Authors:** Christa Hohoff, Tina Kroll, Baoyuan Zhao, Nicole Kerkenberg, Ilona Lang, Kathrin Schwarte, David Elmenhorst, Eva-Maria Elmenhorst, Daniel Aeschbach, Weiqi Zhang, Bernhard T. Baune, Bernd Neumaier, Andreas Bauer, Jürgen Deckert

**Affiliations:** 1 Department of Mental Health, University of Münster, Münster, Germany; 2 Institute of Neuroscience and Medicine (INM-2), Forschungszentrum Jülich, Jülich, Germany; 3 Otto Creutzfeldt Center for Cognitive and Behavioral Neuroscience, University of Münster, Münster, Germany; 4 Institute of Aerospace Medicine, German Aerospace Center, Cologne, Germany; 5 Institute for Occupational and Social Medicine, Medical Faculty, RWTH Aachen University, Aachen, Germany; 6 Division of Sleep Medicine, Harvard Medical School, Boston, MA USA; 7 Department of Psychiatry, Melbourne Medical School, University of Melbourne, Parkville, VIC Australia; 8 The Florey Institute of Neuroscience and Mental Health, The University of Melbourne, Parkville, VIC Australia; 9 Institute of Neuroscience and Medicine (INM-5), Forschungszentrum Jülich, Jülich, Germany; 10 Department of Psychiatry, Psychosomatics and Psychotherapy, Center of Mental Health, University Hospital of Würzburg, Würzburg, Germany

**Keywords:** Psychiatric disorders, Molecular neuroscience, Genetics

## Abstract

Adenosine, its interacting A_1_ and A_2A_ receptors, and particularly the variant rs5751876 in the A_2A_ gene *ADORA2A* have been shown to modulate anxiety, arousal, and sleep. In a pilot positron emission tomography (PET) study in healthy male subjects, we suggested an effect of rs5751876 on in vivo brain A_1_ receptor (A_1_AR) availability. As female sex and adenosinergic/dopaminergic interaction partners might have an impact on this rs5751876 effect on A_1_AR availability, we aimed to (1) further investigate the pilot male-based findings in an independent, newly recruited cohort including women and (2) analyze potential modulation of this rs5751876 effect by additional adenosinergic/dopaminergic gene variation. Healthy volunteers (32/11 males/females) underwent phenotypic characterization including self-reported sleep and A_1_AR-specific quantitative PET. Rs5751876 and 31 gene variants of adenosine A_1_, A_2A_, A_2B_, and A_3_ receptors, adenosine deaminase, and dopamine D_2_ receptor were genotyped. Multivariate analysis revealed an rs5751876 effect on A_1_AR availability (*P* = 0.047), post hoc confirmed in 30 of 31 brain regions (false discovery rate (FDR) corrected *P* values < 0.05), but statistically stronger in anxiety-related regions (e.g., amygdala, hippocampus). Additional effects of *ADORA1* rs1874142 were identified; under its influence rs5751876 and rs5751876 × sleep had strengthened effects on A_1_AR availability (*P*_both_ < 0.02; post hoc FDR-corrected *P*s < 0.05 for 29/30 regions, respectively). Our results support the relationship between rs5751876 and A_1_AR availability. Additional impact of rs1874142, together with rs5751876 and sleep, might be involved in regulating arousal and thus the development of mental disorders like anxiety disorders. The interplay of further detected suggestive *ADORA2A* × *DRD2* interaction, however, necessitates larger future samples more comparable to magnetic resonance imaging (MRI)-based samples.

## Introduction

Anxiety is a fundamental emotion eliciting considerable reactions like increased attention, heart rate, and energy metabolism^1^. In case of pathological anxiety/anxiety disorders, dysregulation in brain regions of the “fear network” occurs, e.g., hyperactivation of amygdala, hippocampus, thalamus, and cingulum, as well as decreased interconnectivity between these regions^2,3^. Anxiety and arousal are tightly connected, both counteract sleep, and both are strongly linked to the adenosinergic system^4–7^. The purine nucleoside adenosine is ubiquitous in body and brain and involved in the regulation of e.g., heart rate, energetic homeostasis, sleep, arousal, and anxiety^4,5,8^. It acts through the four G-protein coupled adenosine receptors (AR) A_1_AR, A_2A_AR, A_2B_AR, and A_3_AR, of which particularly the first is widely distributed in the brain and has a high affinity to adenosine^4–6,9^. An increase in A_1_AR function was reported to elicit anxiolytic effects^10,11^ and quantification of A_1_AR availability via positron emission tomography (PET) demonstrated an A_1_AR increase after sleep loss^12,13^. A further PET investigation revealed a modulation of A_1_AR availability by caffeine^14^, which antagonistically binds to both A_1_AR and A_2A_AR^15^. Both receptors are coexpressed, form heteromers, and interact to regulate adenosinergic and glutamatergic neurotransmission in the brain, which, apart from various physiological functions, have been implicated in pathological alterations related to psychiatric and anxiety disorders (e.g.,^4–6,16–18^). Genetic loss of A_1_AR or A_2A_AR enhanced anxiety-related behavior in knockout mice^19,20^ and variants in the corresponding human genes *ADORA1* and *ADORA2A* were reported to be associated with, for example, sympathetic arousal-related blood-injury phobia^21^, panic disorder^22,23^, and anxiety-related autism spectrum disorder^24^. Further associations were identified in healthy subjects with caffeine-induced anxiety^25–27^ and caffeine effects on sleep (e.g.,^28^). In all these studies *ADORA2A* was reported to have the stronger impact compared with *ADORA1*. Especially the *ADORA2A* single-nucleotide variant rs5751876 (1976C/T, formerly 1083 C/T) revealed strongest and most consistent associations, and its rare T-allele repeatedly turned out at risk for elevated or pathological anxiety (e.g.,^21–23,25–27^). Further studies support the notion that there is a relation between anxiety and caffeine challenge in female rs5751876 TT homozygotes, indicating also a female sex influence in increased anxiety^29,30^.

Among this direct *ADORA2A* rs5751876 effect on anxiety, a previous pilot study reported the T-allele to be associated with increased brain A_1_AR availability in healthy participants as measured by PET^31^, suggesting indirect effects on anxiety via A_1_AR modulation. Although this finding highlights the potential putative role of an interaction between both receptors, the pilot study was restricted to a small sample of 28 male individuals only, allowing only exploratory analyses of few *ADORA2A* and *ADORA1* variants. However, there is evidence to suggest that female sex and additional adenosinergic or dopaminergic genetic variants might as well have a modulatory impact on the rs5751876 effect on A_1_AR availability. Female sex is associated with a higher risk of anxiety/anxiety disorders as well as with a higher risk of sleep- (disruption-) related disorders like insomnia or hypertension (e.g.,^29,32^). A_2B_AR, A_3_AR, and the adenosine deaminase as members of the adenosinergic system mediate adenosine actions via receptor-receptor interactions^33^ and were linked to anxiety- or arousal-related behavior in mice^9,34,35^. The dopamine D_2_ receptor (D_2_R) functions as major interaction partner of A_2A_AR as demonstrated by their colocalization, heteromerization, and cross-desensitization^36,37^. Genetic interaction of *DRD2*-*ADORA2A* variants has been shown to affect caffeine-induced anxiety^26^, whereas *DRD2* variants alone, notably the *DRD2/ANKK1* variant rs1800497 (Taq1A polymorphism), were reported to be associated with anxiety symptoms/disorders (e.g.,^38–41^).

Taken together, previous studies suggest that female sex and additional genetic variations might further modulate the *ADORA2A* rs5751876 effect on brain A_1_AR availability. Therefore, the present study aimed to expand on the original pilot study findings by analyzing a larger set of potentially interacting genetic variants in an independent newly recruited male/female mixed cohort. We hypothesized firstly, that the pilot male-based rs5751876 effect on in vivo brain A_1_AR availability^31^ can be observed also in an independent male-female based sample of healthy humans. Second, we hypothesized that genetic variants of A_1_AR, A_2B_AR, A_3_AR, ADA, and D_2_R contribute to the modulation of the rs5751876 effect on in vivo brain A_1_AR availability.

## Subjects and methods

### Subjects

All subjects were newly recruited via advertisement and a subsequent interview by phone. Forty-three healthy volunteers were finally included in the study (mean age 34.2 ± 14.1 y; 32 males, 11 females; for further details see supplemental material) after obtaining written informed consent for imaging procedure, blood sampling, and genetic testing. Exclusion criteria were neurological or psychiatric disorders, head trauma, systemic diseases, or substance/drug abuse interfering with ARs. All these subjects were phenotypically characterized by a further standardized interview (subjects demographics/characteristics in Table 1) and underwent medical examination before PET imaging at the PET laboratory of the Institute of Neuroscience and Medicine (Forschungszentrum Jülich). All subjects had a history free of psychiatric and neurological diseases. Self-reported habitual sleep duration, current medication, presence of allergies, and consumption of cigarettes, alcohol, coffee and caffeine-containing drinks were obtained by self-rating on a standardized questionnaire routinely used in the PET laboratory (for answers/results see Table 1), which all subjects filled-in before PET imaging. Average sleep duration per night was given in hours and coffee consumption in cups/day (0.15 mL). Whole amount of caffeine-containing drinks per day was assessed in liters. Prior to PET scanning participants were asked to avoid caffeine consumption for at least 24 h. All procedures were approved by the Ethics Committee of the Medical Faculty of the University of Duesseldorf and the German Federal Office for Radiation Protection.

**Table 1:** Participants’ demographics/characteristics in present sample (*N* = 43) and confounding effects on A_1_AR availability and across *ADORA2A* genotype groups.

Demographics/characteristics	*N*	Range	Mean (±SD)	*P* value^6^	A_1_AR availability higher^6^ in:	*P* value^7^
Sex (male/female)	32/11	–	–	<0.05	Females	<0.05
Age (years)	43	18–67	34.22 (14.05)	<0.05	Younger subj.	n.s.
Body height (cm)	43	154–195	177.47 (8.09)	n.s.		n.s.
Body mass index (BMI)	43	18.4–36.2	24.44 (3.63)	n.s.		n.s.
History of disease^1^ (y/n)	7/36	–	–	n.s.		n.s.
Current medication^2^ (y/n)	6/37	–	–	n.s.		n.s.
Allergies^3^ (y/n)	12/27	–	–	<0.05	Affected subj.	n.s.
Sleep duration (h)	43	5.5–8.5	7.35 (0.76)	<0.05	subj. with more sleep	<0.05
Coffee consumption^4^ (y/n)	19/19	–	–	n.s.		n.s.
Caffeine consumption^4^ (y/n)	30/8	–	–	n.s.		<0.05
Alcohol consumption^5^ (y/n)	20/14	–	–	n.s.		<0.05
Cigarette consumption (y/n)	9/34	–	–	n.s.		n.s.
Application mode (bolus/infusion)	8/35	–	–	<0.05	Infused subj.	n.s.
Injected activity (MBq)	43	142–304	244.70 (45.92)	<0.05	subj. with less inj. act.	n.s.
Amount of substance (nmol)	43	0.73–7.67	2.88 (1.65)	<0.05	subj. with less amount	n.s.
Specific activity at scantime (GBq/µmol)	43	28.7–227.7	111.32 (54.20)	n.s.		n.s.

*SD* standard deviation, *y/n* yes/no, *n.s.* not significant (*P* > 0.05), *subj.* subjects, *inj.* injected, *act.* activity.

^1^Including hypertonia, pancreatitis, benign prostatic hyperplasia, sarcoidosis, or psoriasis.

^2^Medications were Metoprololsuccinat, Cadiovan/Ibuprofen, Concor 5 Plus, Pangrol, Teracid, or Timo Stulln 0.5%.

^3^Data missing for four subjects.

^4^“Coffee” stands for coffee drinks alone while “Caffeine” sums drinks of coffee, cola and black tea, data missing for five subjects.

^5^Data missing for nine subjects.

^6^Check for potential confounding effect on brain region specific A_1_AR availability.

^7^Check for potential confounding effects across genotype groups.

### Imaging procedures

PET acquisitions and high-resolution three-dimensional T1-weighted magnetic resonance (MR) images were performed as previously reported^31^ while postprocessing for calculations of ^18^F-8-cyclopentyl-3-(3-fluoropropyl)-1-propylxanthine ([^18^F]CPFPX^42^) binding in cerebral tissues differed slightly. In brief, realignment, coregistrations, segmentation, and normalization of three-dimensional PET and corresponding MRI data were done with PMOD software (version 3.408, PMOD Group, Zürich, Switzerland). For definition of regions of interest, the AAL-template^43^ implemented in the PMOD software was used. Regional time-activity curves (TACs) were corrected for decay and the contribution of intracerebral blood volume. Corrected TACs were used to estimate the A_1_AR availability in terms of the [^18^F]CPFPX binding potential (*BP*_ND_^44^) via the Logan Plot (t* = 20 min, fixed *k*2’^45^) with the cerebellar gray matter as a reference region^46^. Imaging procedures are further detailed in Table 1 and in the supplements. Investigated regions are given in Table 2.

**Table 2:** *ADORA2A* rs5751876 effects on human in vivo A_1_AR availability in a mixed male/female sample.

	Model 1:		Model 2 under modulation of *ADORA1* rs1874142:			
	rs5751876 effect		rs5751876 effect		rs5751876 × sleep effect	
	(Multivariate *P* = 0.047),		(Multivariate *P* = 0.018),		(Multivariate *P* = 0.020),	
	Post hoc ANCOVAs:		Post hoc ANCOVAs:		Post hoc ANCOVAs:	
Human brain region	*P* values	*P*_corr_^1^	*P* values	*P*_corr_^1^	*P* values	*P*_corr_^1^
**Precentral gyrus**	0.014	0.021	**0.0048**	0.025	**0.0059**	0.030
**Rolandic operculum**	**0.002**	0.025	**0.0043**	0.033	**0.0049**	0.038
**Supplementary motor area**	**0.008**	0.029	**0.0053**	0.021	**0.0060**	0.027
Olfactory cortex	0.012	0.025	0.0225	0.030	0.0240	0.032
Superior frontal gyrus	0.011	0.025	0.0114	0.024	0.0130	0.027
**Middle frontal gyrus**	0.015	0.022	**0.0091**	0.024	0.0109	0.028
**Inferior frontal gyrus**	0.011	0.024	**0.0040**	0.041	**0.0047**	0.049
**Gyrus rectus**	**0.007**	0.031	0.0144	0.025	0.0153	0.026
Insula	0.018	0.023	0.0205	0.029	0.0219	0.034
Anterior cingulate cortex	0.035	0.038	0.0518	n.s.	0.0478	0.049
Middle cingulate cortex	0.034	0.037	0.0469	0.050	0.0463	0.049
**Posterior cingulate cortex**	**0.010**	0.027	0.0176	0.027	0.0201	0.033
**Hippocampus/Parahippocampus**	**0.003**	0.022	**0.0047**	0.029	**0.0050**	0.031
**Amygdala**	**0.009**	0.029	**0.0070**	0.024	**0.0081**	0.028
**Calcarine fissures**	**0.001**	0.040	**0.0003**	0.009	**0.0004**	0.012
**Cuneus**	**0.008**	0.031	**0.0048**	0.021	**0.0063**	0.024
Lingual gyrus	0.027	0.032	0.0271	0.034	0.0297	0.037
Occipital lobe	0.013	0.025	0.0136	0.025	0.0148	0.027
Fusiform gyrus	0.015	0.021	0.0107	0.026	0.0114	0.027
Postcentral gyrus	0.031	0.036	0.0302	0.035	0.0341	0.039
Supramarginal gyrus	0.016	0.021	0.0252	0.033	0.0301	0.036
Angular gyrus	0.018	0.022	0.0193	0.028	0.0230	0.032
**Precuneus**	0.013	0.023	**0.0079**	0.024	**0.0097**	0.027
**Paracentral lobule**	**0.002**	0.036	**0.0017**	0.026	**0.0021**	0.033
Caudate	0.013	0.022	0.0285	0.034	0.0285	0.037
Putamen	0.047	0.049	0.0363	0.040	0.0406	0.045
Pallidum	0.215	n.s.	0.2886	n.s.	0.2732	n.s.
**Thalamus**	**0.004**	0.022	0.0120	0.023	0.0122	0.027
Heschl gyrus	0.013	0.024	0.0172	0.028	0.0224	0.033
Parietal	0.010	0.026	0.0112	0.025	0.0130	0.025
**Temporal lobe**	**0.005**	0.028	**0.0082**	0.023	**0.0091**	0.028

Bold face indicates highly significant *P* values (*P* < 0.01); *n.s.* not significant.

^1^Benjamini-Hochberg FDR correction for testing 31 brain regions.

### Genotyping

DNA from blood samples was isolated and used for genotyping of selected variants as described previously^31^ and in detail in the supplements (text and Tabs. S1 and S2). In brief, *ADORA2A* and *ADORA1* variants were the same as used for the pilot sample based on the initially described selection process^31^ (using tagging information of the international HapMap Project, functional potential information of the UCSC Genome browser and association information of previous studies^21–30^). Additional pairwise linkage disequilibrium (LD) analyses performed in the pilot study sample provided LD information and redundancy of several variants^31^. These were dropped, leaving five *ADORA2A* variants (rs5751862, rs5760405, rs2236624, rs5751876, rs4822492) and seven *ADORA1* variants (rs1874142, rs10920568, rs12135643, rs3766566, rs17511192, rs6677137, rs3753472) for the present study (for all see Suppl. Tab S1). All other adenosinergic/dopaminergic variants were selected based on association studies revealing significant variant effects on anxiety or psychiatric disorder-related context, or selected based on functional potential, tagging and frequency information (study references and variant details in Suppl. Tab. S1): rs758857, rs2535609 (A_2B_AR gene *ADORA2B*); rs1890245, rs35254520, rs2786995, rs10776727, rs1544224, rs2229155 (A_3_AR gene *ADORA3*); rs73598374, rs427483 (ADA gene *ADA*); rs4648317, rs7131056, rs4936272, rs4245146, rs17529477, rs55900980, rs1076560, rs6275, rs6277, rs1800497 (D_2_R gene *DRD2*). Of these additional ten adenosinergic and ten dopaminergic gene variants no LD analyses existed so far. Genotypes were determined and controlled for genotyping errors by standard PCR-based methods blind with respect to the phenotypic characteristics of the subjects (for description, primers, and assay conditions see supplements (text and Tab. S2)).

### Statistical analyses

Hardy-Weinberg equilibrium and pairwise LD were assessed using Haploview v.4.1 as reported before^31^. Genotypes were grouped separately for each variant into carriers of both common alleles (common homozygotes) vs. carriers of at least one rare allele (heterozygotes + rare homozygotes); see supplements (Tab. S1). All other analyses were conducted two-sided using SPSS v.25 (IBM, Chicago, IL, USA).

According to our first hypothesis the impact of *ADORA2A* rs5751876 (CC homozygotes vs. T-allele carrier; as previously reported^31^) on A_1_AR availability was investigated in our male-female-based sample in a first analysis model. We started with testing the A_1_AR availability data for mutual dependence (intercorrelation) between brain regions and with testing the participants’ demographics/characteristics for potential confounding, both as reported before^31^ and as detailed in Table 1 as well as in the supplements. Based on the detected intercorrelation and confounders (see results section below) we then utilized general linear model (GLM) multivariate procedure for simultaneous analysis of multiple (intercorrelated) dependent variables^47^ (i.e., the brain region specific A_1_AR availabilities) by genotype group effect with additional confounders in a single model. Thereby the multivariate *ADORA2A* rs5751876 genotype group effect on global A_1_AR brain availability was analyzed and, if significant, followed by post hoc ANCOVAs to identify single brain regions contributing to the global effect as detailed in the supplements. Significance level alpha was set to 0.05 for all pretests and the global multivariate analysis. The ANCOVAs were further corrected for testing 31 brain regions by controlling the false discovery rate (FDR) following the Benjamini-Hochberg procedure^48^.

According to our second hypothesis the *ADORA2A* rs5751876 effect on A_1_AR brain availability was investigated under the assumption of additional modulatory effects of adenosinergic or dopaminergic gene variation. In analogy to the first model, GLM multivariate procedures were used to analyze the rs5751876 genotype group effect on A_1_AR availability (same confounders and model composition), but now including one by one each genetic variant as main factor and in interaction with rs5751876. Significance level was adjusted to 0.025 because of the high similarity to the first model. In case of a significant global effect of rs5751876 on A_1_AR availability, again FDR-corrected post hoc ANCOVAs followed to identify single brain regions contributing to this effect. Testing two to seven genetic variants per gene (*ADORA1*: 6, *ADORA2B*: 2, *ADORA3*: 6, *ADA*: 2, *DRD2*: 7) remained uncorrected in the second model approach.

## Results

All genotype distributions were in Hardy-Weinberg equilibrium (*P* > 0.05). Several variants in close/complete LD (*D*’ = 1.0 and *r*^2^ > 0.8: rs4822492, rs3766566, rs1076560, rs55900980, rs4936272; supplemental Tab. S3) were excluded from further analysis to avoid redundancy. All *ADORA2A* variants revealed high pairwise LD (*D*’ ≥ 0.78), thus potential confounding across individual genotype groups was considered relevant for the whole *ADORA2A* gene and included as confounder for our rs5751876-based analyses.

### *ADORA2A* rs5751876 effect on A_1_AR availability in mixed male/female sample

High intercorrelation of A_1_AR availability between brain regions was detected (all *P* < 0.004, pairwise correlation coefficients: mean = 0.832, range = 0.431–0.983). Analysis of participants’ demographics/characteristics revealed the following statistically relevant confounders: sex, age, self-reported habitual sleep, self-reported presence of allergies, self-reported caffeine and alcohol consumption, application mode of radiotracer, injected activity of radiotracer, and total amount of injected substance (*P*_all_ < 0.05; Table 1). Since sex and sleep had effects on both, A_1_AR availability and across *ADORA2A* genotype groups, additional interaction terms were formed (rs5751876 × sex; rs5751876 × sleep) and further included as confounders.

**Fig. 1:**
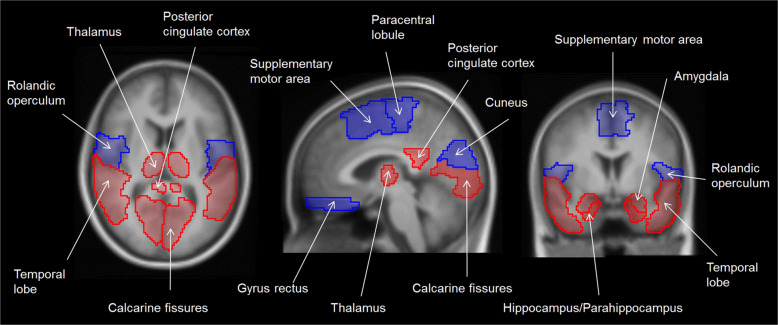
Brain regions with strong *ADORA2A* rs5751876 effects on A_1_AR availability. Mean in vivo MR image illustrates the strongly rs5751876 associated brain regions (post hoc *P* values < 0.01), which are overlaid based on the utilized regional atlas in transversal (left), sagittal (middle), and coronal (right) direction. Red labeling indicates anxiety-related regions and blue labeling the regions without strong relationship to anxiety or anxiety disorders.

Our first analysis model comprising these confounders and interaction terms revealed a significant global multivariate effect of rs5751876 on A_1_AR availability (*P* = 0.047). Post hoc ANCOVAs confirmed this effect for nearly all (30 of 31) investigated brain regions with nominal *P* values even robust to Benjamini-Hochberg FDR correction for testing multiple regions (corrected *P*_all_ < 0.05 (except pallidum); Table 2). Moreover, anxiety-related regions such as amygdala, hippocampus–parahippocampus, cingulate cortex, calcarine fissures, temporal lobe, and thalamus were found strongly associated with nominal *P* values < 0.01 (Fig. 1; Table 2).

### Modulatory influence of *ADORA1* rs1874142 on rs5751876 and rs5751876 × sleep effects

**Fig. 2:**
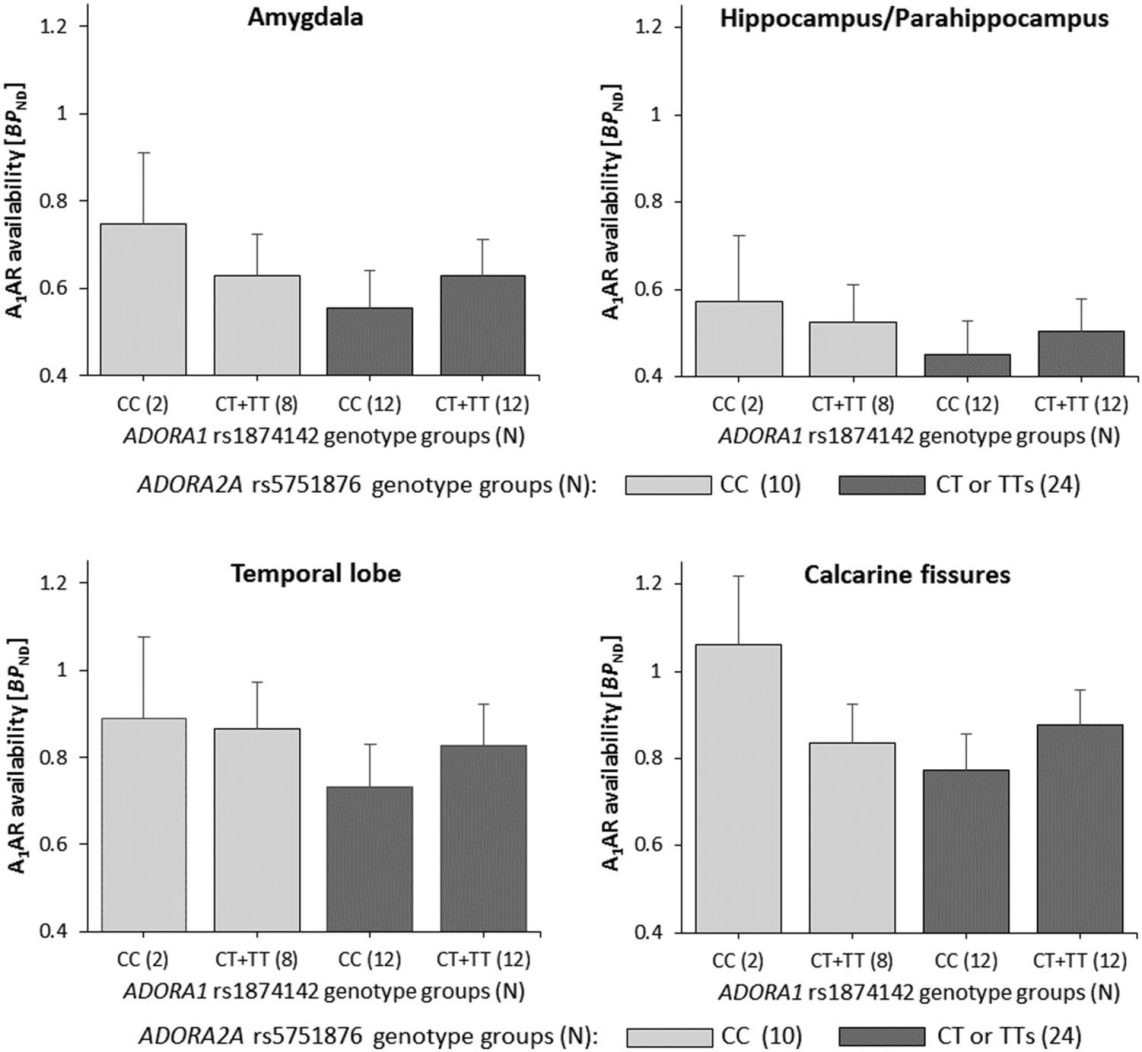
Modulatory influence of *ADORA1* rs1874142 on *ADORA2A* rs5751876 effect. GLM multivariate bar plots based on second model post hoc analyses illustrate significant rs5751876 effect on A_1_AR availability in the anxiety-related regions amygdala (*P* = 0.007; FDR *P*_corr_ = 0.024), hippocampus/Parahippocampus (*P* = 0.005; FDR *P*_corr_ = 0.029), temporal lobe (*P* = 0.008; FDR *P*_corr_ = 0.023), and calcarine fissures (*P* = 0.0003; FDR *P*_corr_ = 0.009) under the modulatory influence of the *ADORA1* variant rs1874142. Plots represent adjusted means according to sleep (7.37 h), age (33.4 y), injected activity (240.06 MBq), and amount of substance (2.91 nmol) with standard error bars (SEM).

Second model analyses revealed *ADORA1* rs1874142 to statistically strengthen the model, i.e. under the influence of rs1874142 stronger significances were detected for the effects of rs5751876 (global *P* = 0.018; Table 2) and the interaction rs5751876 × sleep (global *P* = 0.020) on A_1_AR availability. Both these rs5751876-related terms revealed *P* values ranging below the adjusted significance level of 0.025. Further, both findings could be confirmed post hoc for nearly all investigated brain regions (Table 2; FDR-corrected *P* values < 0.05 (except pallidum and anterior cingulum for rs5751876; except pallidum for rs5751876 × sleep)). Again, anxiety-related regions such as amygdala, hippocampus/Parahippocampus, calcarine fissures, and temporal lobe were found strongly associated with nominal *P* values < 0.01 (Fig. 2; Table 2).

In addition, *ADORA2B* rs2535609 seemed to statistically strengthen the model. Under its influence, stronger significances were detected for the multivariate effects of rs5751876 (global *P* = 0.005) and rs5751876-related interactions (rs5751876 × sleep, global *P* = 0.005; rs5751876 × rs2535609, global *P* = 0.012) on A_1_AR availability (Table 3). However, none of these multivariate findings could be confirmed post hoc for individual brain regions, as none of them survived correction for multiple brain region testing (all FDR-corrected *P* values > 0.05).

**Table 3:** Putative modulatory effect of additional adenosinergic or dopaminergic gene variants on the effects of *ADORA2A* rs5751876 on human in vivo A_1_AR availability in a mixed male/female sample.

	Model 2 multivariate global effects (*P* values) of:			Post hoc ANCOVA	
Gene and putative modulatory variant:	rs5751876	rs5751876 × sleep	rs5751876 × modulatory variant	Brain region specific rs5751876 P values	FDR corrected *P* values^1^
***ADORA1***					
**rs1874142**	**0.018**	**0.020**	**0.032**	**<0.05**	**<0.05**
rs10920568	0.639	0.664	0.387	–	–
rs12135643	0.448	0.494	0.577	–	–
rs17511192	0.078	0.082	0.077	–	–
rs6677137	0.121	0.137	0.154	–	–
rs3753472	0.129	0.138	0.084	–	–
***ADORA2B*:**					
rs758857	0.207	0.218	0.327	–	–
**rs2535609**	**0.005**	**0.005**	**0.012**	**<0.05**	n.s.
*ADORA3*:					
rs1890245	0.308	0.407	0.148	–	–
rs35254520	0.071	0.086	0.050	–	–
rs2786995	0.691	0.745	0.461	–	–
rs10776727	0.384	0.432	0.521	–	–
rs1544224	0.255	0.265	0.346	–	–
rs2229155	0.264	0.261	0.193	–	–
*ADA*:					
rs73598374	0.057	0.063	0.102	–	–
rs427483	0.483	0.529	0.730	–	–
*DRD2*:					
rs4648317	0.318	0.346	0.338	–	–
rs7131056	0.578	0.634	0.835	–	–
rs4245146	0.536	0.521	0.390	–	–
rs17529477	0.563	0.601	0.713	–	–
rs6275	0.325	0.360	0.379	–	–
rs6277	0.451	0.512	0.347	–	–
rs1800497	0.642	0.678	0.423	–	–

Bold face indicates significant modulatory effect; *n.s.* not significant.

^1^Benjamini–Hochberg FDR correction for testing 31 brain regions.

All other adenosinergic or dopaminergic gene variants seemed to statistically weaken second model analyses (for all: multivariate global *P* values > 0.05, see Table 3).

## Discussion

Our present study revealed two main findings. First, we were able to corroborate the *ADORA2A* rs5751876 effect on A_1_AR availability, originally detected in our pilot sample of males only^31^, in an independent male/female sample. This allowed a more-generalized interpretation compared with the pilot model and thus further underlined the important role of *ADORA2A* interacting with A_1_AR as detailed in the introduction (e.g.,^4–6,16–18^). Second, our findings showed modulation by *ADORA1* rs1874142, resulting in statistically strengthened effects of rs5751876 and rs5751876 × sleep on A_1_AR availability. In these findings, anxiety-related brain regions were particularly involved, underscoring the close link between *ADORA2A* rs5751876, anxiety, and anxiety disorders. This is in line with the variety of anxiety-relevant investigations reported in the introduction (e.g.,^21–27^). The recruitment of slightly more individuals compared with the pilot study appears to have increased the power of the rs5751876 effect enough (28 subjects in pilot vs. 43 in present sample), to have resulted in now statistically significant multivariate results in contrast to the pilot study^31^. Furthermore, the identified interactive effect of rs5751876 × sleep on A_1_AR availability under the *ADORA1* rs1874142 modulation underscores the close interrelationship between the adenosine system, particularly the interplay of A_1_ and A_2A_ receptors, and sleep as mentioned before^5,8,12,13,28^.

However, though the rs5751876 effect on A_1_AR availability was distinctly stronger in the present sample compared with the pilot study, the direction of the rs5751876 effect disclosed discrepancies. In the pilot study the rs5751876 T-allele increased A_1_AR availability^31^, whereas in the present sample the T-allele predominantly decreased A_1_AR availability (in 31 regions in the first model; in 23 regions in the second model under modulation of *ADORA1* rs1874142). Analysis of the underlying genotype effects (rs5751876 CC vs. CT vs. TT) uncovered consistent CT to TT (lower to higher A_1_AR availability, respectively) gene-dose effects in both samples. In contrast, the CC carriers scored lowest (with respect to A_1_AR availability) in the pilot, thereby completing the gene-dose distribution, but high in the present sample (in 23 of 31 brain regions even higher than TTs), thereby forming a U-shaped distribution (Suppl. Fig. S1). Though sex differences exist between both samples (males only vs. mixed males/females), female sex was not related to the detected discrepancy in the direction of the rs5751876 genotype effect between present and pilot sample (present sample males alone: similar rs5751876 T-allele coupled decrease in A_1_AR availability in 30 of 31 brain regions). Besides intended sex differences, both samples were similarly recruited with regard to demographic data like age, body mass index, history of diseases, and habits like sleep, coffee, and nicotine consumption.

To probe alternative explanations for this discrepancy in the direction of the rs5751876 genotype effect and based on the strong interaction of rs5751876 × sleep, we checked if sleep habit might contribute to the rs5751876 genotype effect discrepancies between present and pilot study^31^. As both samples show comparable sleep habits (mean ± SD self-reported habitual sleep duration of present sample 7.35 ± 0.76 vs. pilot sample 7.32 ± 1.01; *P* = 0.811). Thus, we dichotomized the variant in the subgroup categories “less sleep than mean” (less sleep) and “more sleep than mean” (more sleep) with comparable subgroup sizes in both samples (*N* (less/more sleep) in present sample: 21/22; in pilot sample: 14/13). In these subgroups, we qualitatively rechecked the respective rs5751876 genotype dependent distribution of A_1_AR availability values with promising findings. In the less sleep group of the pilot sample most regions showed a linear gene-dose relationship, but however 7 of 19 brain regions (hippocampus, thalamus, caudate, putamen, parietal cortex, occipital cortex, sensorimotor cortex; Suppl. Tab. S4) revealed indeed U-shaped distribution, which resembles the U-shaped distribution in 31 brain regions in our present study (Suppl. Tab. S5). In contrast, the more sleep group of the pilot sample revealed the above mentioned gene-dose distribution in almost all (17 of 19) regions (Suppl. Tab. S4; only posterior cingulate gyrus and putamen with inverted U-shaped distributions). This again resembles the more sleep group of our present study revealing as well gene-dose genotype distribution in 13 of 31 brain regions, including particularly the anxiety-relevant regions amygdala, hippocampus/Parahippocampus, cingulate cortex (anterior, middle), thalamus, temporal lobe, calcarine fissures, and occipital lobe; Suppl. Tab. S5). Taken together, though self-reported and not measured by objective methods like actigraphy or EEG, sleep might explain at least several of the observed discrepancies. This is in line with previous findings of a tight connection between *ADORA2A* and sleep as well as between sleep and A_1_AR availabilities. *ADORA2A* variants (including rs5751876) modulate psychomotor vigilance in a rested state and after sleep deprivation^28^, as well as A_1_AR availability increases after prolonged wakefulness^12,13^. Slight A_1_AR availability increases might also occur in our less sleep group participants owing to their shorter habitual sleep duration and therefore slightly prolonged wakefulness. Such slightly increased A_1_AR availability might then have obscured or masked the presumably small effect size of the *ADORA2A* rs5751876 T-allele effect. In contrast, our more sleep group probably would not have slightly prolonged wakefulness and therefore presumably would have overall lower A_1_AR availabilities. In such cases, the small effect of the rs5751876 T-allele and the resulting slightly upregulated A_1_AR availability might be visible. Such slight but possibly permanent upregulation of A_1_ARs by genotype might provide protection against overly increased arousal or anxiety, or even anxiety disorders. Indeed a protective role of upregulated A_1_AR was reported toward hyperarousal associated with restless legs syndrome^49^. In rodents, upregulation or positive allosteric modulation of A_1_AR caused robust anxiolytic effects^10,11^, whereas lack of A_1_AR enhanced anxiety and arousal^20^.

Finally, we checked all 31 additional adenosinergic and dopaminergic gene variants for potentially divergent genotype group distributions in pilot vs. present sample by nonparametric statistics (Fisher’s exact test; see also Suppl. Tab. S6 with footnotes), which might further explain the rs5751876 genotype effect discrepancies between both samples. Strikingly, *DRD2* variants rs4245146 and rs1800497 were distributed differently between both samples (both *P* < 0.05; Suppl. Tab. S6), which might contribute to the gene-dose vs. U-shaped rs5751876 effects. Although 15 rs4245146 CC homozygotes existed in the present sample, only one was present in the pilot (Suppl. Tab. S6), and this rs4245146 CC-homozygosity seemed to further modulate *ADORA2A* rs5751876 effects. The 15 rs4245146 CC homozygotes had increased A_1_AR availabilities in all 31 brain regions in rs5751876 CC carriers (11 regions *P* < 0.05; 10 regions *P* < 0.1) but had decreased A_1_AR availabilities in all 31 brain regions in rs5751876 CT-/TT-carriers (one region *P* < 0.05; one region *P* < 0.1; Suppl. Tab. S7). Thus, rs4245146 CC-dependent modulation might interplay with the rs5751876 effect on A_1_AR availability in the present sample (Suppl. Fig. S2) but not the pilot sample with only one rs4245146 CC-subject. As well, carriers of the rare rs1800497 A-allele are four times more frequent in the present sample (*N* = 16) than in the pilot (*N* = 4; Suppl. Tab. S6). This higher number of A-allele carriers might have lowered individual D_2_R levels, in line with a reduced striatal D_2_R binding already shown in rs1800497 A-allele carriers^50^. As A_2A_ and D_2_ receptors have a tight spatial/functional interplay and interact antagonistically^36,37^, lower D_2_R levels could lead to increased A_2A_R levels in our 16 rs1800497 A-allele carriers. Via the similarly tight spatial/functional antagonistic interplay of A_2A_ and A_1_ receptors (c.f. introduction), such putatively increased A_2A_R levels in the 16 subjects (=37% of present sample) might have decreased A_1_AR levels and thereby covered or obscured the presumably mild *ADORA2A* rs5751876 effect on A_1_AR availability. In comparison, Eisenstein et al.^50^ reported normal striatal D_2_R specific binding in rs1800497 GG-homozygotes, which might be the precondition to clearly identify the small effect size *ADORA2A* rs5751876 genotype group effects in our present study (63% of subjects vs. 86% of pilot subjects). Interestingly, both *DRD2* variants, rs1800497 and rs4245146, were described to play prominent roles in anxiety before (e.g.,^39–41^), further supporting our more distinctive findings in anxiety-related brain regions. Moreover, dopaminergic genes including *DRD2* have been shown to be involved in inverted U-shaped relationships between dopamine signaling and prefrontal cortical function (e.g.,^51,52^), supporting our assumption of *DRD2* variants presumably contributing to our sample *ADORA2A* rs5751876 differences. Unfortunately, the size of our present sample did not allow inclusion of additional genetic variants like rs4245146 or rs1800497 together with rs5751876 and rs1874142, as the model is underpowered then, warranting distinctly larger samples in subsequent investigations.

A next step could be to compare individuals with and without anxiety symptoms and heart rate measurements with regard to their objectively measured sleep duration and genotypes. Such multidimensional approach would demand higher statistical power, which underscores the need to extend future sample size. In our present study it was modest, albeit in the range of comparable recent PET-based studies (e.g.,^13,53,54^) and well-powered enough for our *ADORA2A* rs5751876-focused analyses to detect the hypothesized effects. It did unfortunately not allow for additional detailed investigation of for example additional interacting effects of *DRD2* variants together with *ADORA2A* rs5751876 and *ADORA1* rs1874142 in the same multivariate models as mentioned above. Further, our study provided only cross-sectional data, whereas a longitudinal study might allow prospective insights and maybe the potential for interventional approaches.

## Conclusion

Our present findings support the role of *ADORA2A* rs5751876 on in vivo A_1_AR availability in the human brain. Further analyses suggested additional modulatory effects of *ADORA1* rs1874142 on the effects of rs5751876 and rs5751876 × sleep on A_1_AR availability. Together, these effects might contribute to the regulation of the sleep-arousal system and thus might be involved in the development of particularly arousal-related mental disorders such as anxiety disorders. However, transferability of our results between present and pilot samples was difficult due to small effect sizes and complex gene × gene (*ADORA2A* × *ADORA1*, *ADORA2A* × *DRD2*) and gene × environment (*ADORA2A* × sleep) interactions, which modulated the direction of the biological outcome, i.e., A_1_AR availability in the brain. This might complicate also comparisons with future samples and therefore necessitates increased sample sizes, more comparable to current MRI-based samples, to allow the detection of such complex underlying and modifying relationships. In the next steps, more in-depth analyses of the *ADORA2A* rs5751876 × sleep relation are required based on objectively measured individual sleep parameters ideally complemented by more-detailed information of individual anxiety sensitivity, life styles, (night shift) workload, and past or present stressful life events. Further, investigation of samples preferably including also individuals with anxiety symptoms and in a longitudinal design are needed.

## Supplementary information

Supplemental Material
