# Supplementary material for: ADORA2A variation and adenosine A1 receptor availability in the human brain with a focus on anxiety-related brain regions: modulation by ADORA1 variation
Source: Transl Psychiatry. 2020 Nov 24;10:406. doi: 10.1038/s41398-020-01085-w (PMC7686488; doi:10.1038/s41398-020-01085-w)
Supplement: Supplementary file 1 — Supplemental Material [file 41398_2020_1085_MOESM1_ESM.doc]

***ADORA2A* variation and adenosine A_1_ receptor availability in the human brain with a focus on anxiety-related brain regions: Modulation by *ADORA1* variation**

Christa Hohoff^1*$^, Tina Kroll^2$^, Baoyuan Zhao^1^, Nicole Kerkenberg^1,3^, Ilona Lang^1,3^, Kathrin Schwarte^1^, David Elmenhorst^2^, Eva-Maria Elmenhorst^4,5^, Daniel Aeschbach^4,6^, Weiqi Zhang^1,3^, Bernhard T. Baune^1,3,7,8^, Bernd Neumaier^9^, Andreas Bauer^2^, Jürgen Deckert^10^

#### ^1^Department of Mental Health, University of Münster, Germany; ^2^Institute of Neuroscience and Medicine (INM-2), Forschungszentrum Jülich, Germany; ^3^Otto Creutzfeldt Center for Cognitive and Behavioral Neuroscience, University of Münster, Germany; ^4^Institute of Aerospace Medicine, German Aerospace Center, Cologne, Germany; ^5^Institute for Occupational and Social Medicine, Medical Faculty, RWTH Aachen University, Germany; ^6^Division of Sleep Medicine, Harvard Medical School, Boston, MA, USA; ^7^Department of Psychiatry, Melbourne Medical School, University of Melbourne, VIC, Australia; ^8^The Florey Institute of Neuroscience and Mental Health, The University of Melbourne, Parkville, VIC, Australia; ^9^Institute of Neuroscience and Medicine (INM-5), Forschungszentrum Jülich, Germany; ^10^Department of Psychiatry, Psychosomatics and Psychotherapy, Center of Mental Health, University Hospital of Würzburg, Germany

^$^ These authors contributed equally as joint first authors

**Supplementary information:**

**Subjects and methods:**

***Subjects.*** Subjects enrolled in this study were recruited in different cohorts at varying time-points and with different foci (e.g. one cohort with only females, cohort with older men only etc.). Specific activities and hence following injected amounts of substance as well as injected activities (lower for females) and age varied significantly for the different cohorts of subjects (Kruskal-Wallis test p<0.05). In addition, comorbidities and concomitant medication agglomerated in the cohort of older men, which were coincidentally injected with higher amounts of substance of radiotracer.

For our present study (combining all cohorts, N=43), the detected statistically relevant confounding of injected activity of radiotracer and total amount of injected substance on A_1_AR availability was likely due to the above mentioned co-varying factors like e.g. age, sex and comorbidities between subjects scanned in one cohort during a certain time-period. Individual correlation analysis per cohort for relationship of injected amount of substance or injected activity of radiotracer respectively with A_1_AR availability revealed no significant dependencies as expected.

Results gained by parts of the subjects were already published elsewhere [13,51], but none of these subjects were part of our pilot study [31]. For subjects being included in Elmenhorst et al. ([13]; N=14, males), A_1_AR availability detected in the scan after recovery sleep was analyzed in the current study. In these 14 male subjects, A_1_AR availabilities after recovery sleep were similar compared to baseline A_1_AR availabilities of the other N=29 subjects of the present study (P-values per brain region all >0.21). Both subgroups were thus combined to increase statistical power for subsequent multivariate analyses (see below).

***Imaging procedures.*** 3D PET was acquired on a Siemens ECAT EXACT HR+ scanner (Siemens CTI, Knoxville, TN, USA) for 90-140 min starting simultaneously with the injection of 8-cyclopentyl-3-(3-[18F]fluoropropyl)-1-propylxanthine ([^18^F]CPFPX) being synthesized in-house as described previously [52]. For bolus-infusion studies the kbol (amount of bolus equaling an infusion of a certain lengths) was between 55 and 63 min. Arterialized venous blood samples were taken on following time points after start of emission scan: 1, 5, 10 min and every 10 min afterwards for subsequent blood volume correction of brain regions of interest (ROIs) by correcting brain time activity curves (TACs) for 5% of total blood activity.

Realignment, coregistrations, segmentation and normalization of three-dimensional PET and corresponding MRI data (MPRAGE sequence acquired on a 3T Siemens Magnetom Trio, oriented to the anterior/ posterior commissure line) was done with PMOD software (version 3.408, PMOD Group, Zürich, Switzerland). For definition of regions of interest the AAL-template [42] implemented in the PMOD software was used with slight adaptations to the cerebellar grey matter as reference region. Regional time-activity curves (TACs), derived from gray matter masked PET images (standard calculation algorithm of probability maps implemented in PMOD software), of the first 60 min of the emission scan were side-averaged, corrected for decay and the contribution of intracerebral blood volume. Corrected TACs were used to estimate regional A_1_AR availability in terms of the binding potential (*BP*_ND_) – a parameter directly proportional to the respective receptor availability [53] – via the Logan Plot [43] with *t** = 20 min, a fixed *k*2’ of 0.15/min and the cerebellar grey matter as a reference region as described previously [44].

***Genotyping.*** DNA from blood samples (N=43 biological specimen, one per subject) was extracted and used for genotyping as described previously in the pilot study [31]. Briefly, DNA was isolated from blood samples of included study subjects using standard methods (FlexiGene DNA Kit, Qiagen). Genotyping of the 12 *ADORA1* and *ADORA2A* variants (rs1874142, rs10920568, rs12135643, rs3766566, rs17511192, rs6677137, rs3753472, and rs5751862, rs5760405, rs2236624, rs5751876, rs4822492, respectively) followed polymerase chain reaction (PCR)-based restriction fragment length polymorphism (RFLP) analysis as detailed in [31]. That is, standard PCRs in a total of 20µl comprised 45 ng DNA, 10 pmol per primer, 200 µM dNTPs, 0.4 U Taq Polymerase, and 2.5 mM MgCl_2_. Cycling conditions started with initial 5 min at 94°C (standard polymerase type) or 15 min at 95°C (HotStart polymerase type), followed by 35 cycles (30 s at 94 °C, 30 s at T, 60 s at 72 °C), and a final step of 10 min (72 °C). PCR products were then digested with selected restriction enzymes specific for the variant-alleles, resulting in individual and allele-specific distinguishable RFLP fragments. These were separated via polyacrylamide gels and visualized by silver staining to obtain and analyze subject-specific RFLP fragment banding patterns for genotype assignment.

Genotyping of the 20 other adenosinergic/dopaminergic variants (for a detailed variant list see Suppl. Tab. S1) followed the same PCR-based RFLP-analysis approach as described above and as detailed in Suppl. Tab. S2. That is, standard PCRs of same volumes and contents as before were performed under same cycling conditions, followed again by PCR product digestion with selected allele-specific restriction enzymes, separation on polyacrylamide gels and visualization by silver staining (Tab. S2). We further controlled for possible genotyping errors by independently and directly sequencing >10% (at least two representatives of each genotype; serving as technical replicates) randomized subjects. This revealed always RFLP-identical results and thus concordance rates of 100%. In all cases genotypes were assigned blind that is without knowledge of the phenotypic characteristics of the subjects.

***Statistical Analyses.*** With regard to our first hypothesis assuming successful extension of the *ADORA2A* rs5751876 effect on brain A_1_AR availability in a mixed male/female sample, we utilized our first analysis model, prior to which several pretests were needed. We started with testing the A_1_AR availability data for mutual dependence (intercorrelation) between brain regions as reported before [31]. Briefly, after testing for deviation from normal distribution (one-sample Kolmogorov-Smirnov and Shapiro-Wilk test) revealing normal distribution, we used parametric Pearson’s correlation analysis. Via pair-wise analysis of all 31 brain regions we obtained the degree of correlation and thus an indicator of mutual dependence, which is high in case of high (inter)correlation findings (correlation coefficients > 0.3) between brain regions.

Next the participants’ demographics/characteristics were tested for potential confounding as reported before [31]. Briefly, all continuous data like age, BMI, sleep duration, injected activity of radiotracer, or total amount of injected substance were tested for deviation from normal distribution as described above. In case of normal distribution, parametric tests were used subsequently, otherwise non-parametric tests. All participants’ demographic/characteristic data were then checked for potential confounding of A_1_AR availability by parametric (categorical/dichotome data: Student’s T-Test; continuous data: Pearson’s correlation analysis) or nonparametric (categorical/dichotome data: Mann-Whitney U-test, continuous data: Spearman’s rank correlation analysis) statistics. Potential confounding across individual *ADORA2A* genotype groups was tested using nonparametric statistics (categorical/dichotome data: Pearson’s chi-square test or Fisher’s exact test; continuous data: Mann-Whitney U-test).

On this basis, the first analysis model was then utilized. For the GLM multivariate procedure, all 31 brain region specific A_1_AR availability data were included simultaneously as dependent (multi)variats, which is recommended in case of mutual dependent (highly intercorrelated) variables, so that they were analyzable in a single multivariate model [54]. In addition, all identified statistically relevant confounders together with *ADORA2A* rs5751876 (genotype group) were included in this model (continuous data such as age or sleep as variables, categorical/dichotome data such as sex as factors). The model was based on the assumption of main effects for all included confounders and *ADORA2A* rs5751876 and additional interaction terms if relevant. If multivariate analysis turned out significant, post-hoc ANCOVAs were used to test for brain region-specific effects as implemented in and automatically performed by SPSS.

**References:**

13. Elmenhorst D. *et al.* Recovery sleep after extended wakefulness restores elevated A1 adenosine receptor availability in the human brain. *Proc. Natl. Acad. Sci. USA* **114**, 4243-42480 (2017).

31. Hohoff C. *et al.* Association of adenosine receptor gene polymorphisms and in vivo adenosine A1 receptor binding in the human brain. *Neuropsychopharmacology* **39**, 2989-2999 (2014).

42. Tzourio-Mazoyer N. *et al.* [Automated anatomical labeling of activations in SPM using a macroscopic anatomical parcellation of the MNI MRI single-subject brain.](https://www.ncbi.nlm.nih.gov/pubmed/11771995) *Neuroimage* **15**, 273-289 (2002).

43. Logan J*. et al.* [Distribution volume ratios without blood sampling from graphical analysis of PET data.](https://www.ncbi.nlm.nih.gov/pubmed/8784228) *J. Cereb. Blood Flow. Metab.* **16**, 834-8400( 1996).

44. Meyer PT. *et al.* Effect of aging on cerebral A1 adenosine receptors: A [18F]CPFPX PET study in humans. *Neurobiol. Aging* **28**, 1914-1924 (2007).

51. Elmenhorst EM. *et al.* [Cognitive impairments by alcohol and sleep deprivation indicate trait characteristics and a potential role for adenosine A1 receptors.](https://www.ncbi.nlm.nih.gov/pubmed/30012607) *Proc. Natl. Acad. Sci. USA* **115**, 8009-8014 (2018).

52. Holschbach MH. *et al.* Synthesis and evaluation of no-carrier-added 8-cyclopentyl-3-(3-[(18)F]fluoropropyl)-1-propylxanthine ([(18)F]CPFPX): a potent and selective A(1)-adenosine receptor antagonist for in vivo imaging. *J. Med. Chem.* **45**, 5150-5156 (2002).

53. Innis RB. *et al.* Consensus nomenclature for in vivo imaging of reversibly binding radioligands. *J. Cereb. Blood Flow. Metab.* **27**, 1533-1539 (2007).

54. Field A (ed) *Discovering Statistics Using IBM SPSS Statistics* (SAGE Publications, London, 2009).

**Table S1:** Characteristics of adenosinergic/dopaminergic gene variants *ADORA1*, *-2A*, *-2B*, *-3*, *ADA*, and *DRD2* and variant allele frequencies in own present sample (N=43)

| Variation: | UCSC variation position: | | | Variant allele frequencies: | References: | Variant allele frequencies: |
| --- | --- | --- | --- | --- | --- | --- |
| ID: | Chr.: | Base position: | Functional region: | dbSNP/gnomAD: | PubMed (see reference list), UCSC, dbSNP, 1000genome, gnomAD: | Present sample (genotype groups, N): |
| ***ADORA2A*** |  |  |  |  |  |  |
| rs5751862 | 22 | 24 406 596 | intron | G=0.512, A=0.488 | e.g., Hohoff et al. 2014 | G=0.535, A=0.465; (GG=11, GA/AA=32) |
| rs5760405 | 22 | 24 417 873 | intron | C=0.771, T=0.229 | e.g., Hohoff et al. 2014 | C=0.837, T=0.163; (CC=29, CT/TT=14) |
| rs2236624 | 22 | 24 440 056 | intron | C=0.727, T=0.273 | e.g., Hohoff et al. 2014 | C=0.686, T=0.314; (CC=19, CT/TT=24) |
| **rs5751876** | 22 | 24 441 333 | exon (silent) | C=0.594, T=0.406 | e.g., Hohoff et al. 2014 | C=0.512, T=0.488; (CC=11, CT/TT=32) |
| rs4822492 | 22 | 24 447 626 | downstream/AS1 | G=0.576, C=0.424 | e.g., Hohoff et al. 2014 | G=0.512, C=0.488; (CC=10, CG/GG=33) |
| *ADORA1* |  |  |  |  |  |  |
| rs1874142 | 1 | 203 125 278 | promoter | G=0.614, A=0.386 | e.g., Hohoff et al. 2014 | G=0.616, A=0.384; (GG=16, GA/AA=27) |
| rs10920568 (=rs2228079) | 1 | 203 129 147 | exon | T=0.666, G=0.334 | e.g., Hohoff et al. 2014 | T=0.698, G=0.302; (TT=20, TG/GG=23) |
| rs12135643 | 1 | 203 131 170 | intron | C=0.804, A=0.196 | e.g., Hohoff et al. 2014 | C=0.779, A=0.221; (CC=26, CA/AA=17) |
| rs3766566 | 1 | 203 136 227 | intron | G=0.774, A=0.226 | e.g., Hohoff et al. 2014 | G=0.756, A=0.244; (GG=24, GA/AA=19) |
| rs17511192 | 1 | 203 150 363 | intron | C=0.543, T=0.457 | e.g., Hohoff et al. 2014 | C=0.512, T=0.448; (CC=11, CT/TT=32) |
| rs6677137 | 1 | 203 154 130 | intron | T=0.582, C=0.418 | e.g., Hohoff et al. 2014 | T=0.686, C=0.314; (TT=19, TC/CC=24) |
| rs3753472 | 1 | 203 162 155 | intron | T=0.635, C=0.365 | e.g., Hohoff et al. 2014 | T=0.709, C=0.291; (TT=21, TC/CC=22) |
| *ADORA2B*: |  |  |  |  |  |  |
| rs758857 | 17 | 15 950 943 | intron | A=0.841, G=0.159 | e.g., Figler et al. 2011 | A=0.802, G=0.198; (AA=27, AG/GG=16) |
| rs2535609 | 17 | 15 953 426 | intron | A=0.774, G=0.226 | UCSC/dbSNP/1000genome/gnomAD | A=0.826, G=0.174; (AA=29, AG/GG=14) |
| *ADORA3*: |  |  |  |  |  |  |
| rs1890245 | 1 | 111 516 060 | upstream | A=0.720, T=0.280 | UCSC/dbSNP/1000genome/gnomAD | A=0.674, T=0.326; (AA=19, AT/TT=24) |
| rs35254520 | 1 | 111 515 273 | upstream | C=0.641, G=0.359 | UCSC/dbSNP/1000genome/gnomAD | C=0.581, G=0.419; (CC=13, CG/GG=30) |
| rs2786995 | 1 | 111 507 917 | upstream | C=0.507, G=0.452, T=0.041 | UCSC/dbSNP/1000genome/gnomAD | C=0.488, G=0.465; T=0.047;  (CC=9, CGGG=30; N=4 T-allele carrier) |
| rs10776727 | 1 | 111 504 404 | promoter | A=0.569, C=0.431 | e.g., Kim et al. 2009, 2010; Campbell et al. 2013 | A=0.523, C=0.477; (AA=9, AC/CC=34) |
| rs1544224 | 1 | 111 503 918 | promoter | G=0.732, A=0.269 | e.g., Kim et al. 2009, 2010; Campbell et al. 2013 | G=0.686, A=0.314; (GG=22, GA/AA=21) |
| rs2229155 | 1 | 111 500 010 | exon/UTR | G=0.822, A=0.178 | e.g., Kim et al. 2009, 2010; Campbell et al. 2013 | G=0.756, A=0.244; (GG=24, GA/AA=19) |

To be continued

**Table S1:** continued

| *ADA*: |  |  |  |  |  |  |
| --- | --- | --- | --- | --- | --- | --- |
| rs73598374 | 20 | 44 651 586 | exon | G=0.946, A=0.054 | e.g., Dutra et al. 2010; Hettinger et al. 2008; Bachmann et al. 2012 | G=0.942, A=0.058; (GG=38, GA/AA=5) |
| rs427483 | 20 | 44 651 193 | intron | C=0.725, G=0.275 | UCSC/dbSNP/1000genome/gnomAD | C=0.814, G=0.186; (CC=29, CG/GG=14) |
| *DRD2*: |  |  |  |  |  |  |
| rs4648317 | 11 | 113 460 810 | intron | G=0.847, A=0.153 | UCSC/dbSNP/1000genome/gnomAD | G=0.826, A=0.174; (GG=30, GA/AA=13) |
| rs7131056 | 11 | 113 459 052 | intron | C=0.526, A=0.475 | e.g., Todt et al. 2007; Sipilä et al. 2010 | C=0.523, A=0.477; (CC=12, CA/AA=31) |
| rs4936272 | 11 | 113 448 185 | intron | C=0.601, T=0.399 | UCSC/dbSNP/1000genome/gnomAD | C=0.605, T=0.395; (CC=15, CT/TT=28) |
| rs4245146 | 11 | 113 447 251 | intron | C=0.601, T=0.399 | e.g., Sipilä et al. 2010 | C=0.616, T=0.384; (CC=15, CT/TT=28) |
| rs17529477 | 11 | 113 446 345 | intron | G=0.744, A=0.256 | UCSC/dbSNP/1000genome/gnomAD | G=0.744, A=0.256; (GG=24, GA/AA=19) |
| rs55900980 | 11 | 113 413 864 | intron | G=0.824, Gdel (-)=0.176 | UCSC/dbSNP/1000genome/gnomAD | G=0.802, Gdel=0.198; (GG=28, G---=15) |
| rs1076560 | 11 | 113 412 966 | intron | C=0.823, A=0.177 | e.g., Blasi et al. 2009; Clarke et al. 2014 | C=0.802, A=0.198; (CC=28, CA/AA=15) |
| rs6275 | 11 | 113 412 755 | exon | G=0.694, A=0.306 | e.g., Ghosh et al. 2013 | G=0.756, A=0.244; (GG=26, GA/AA=17) |
| rs6277 | 11 | 113 412 737 | exon | A=0.530, G=0.470 | e.g., Zhang et al. 2007; Betcheva et al. 2009; Hirvonen et al. 2009; Whitmer and Gotlib 2012 | A=0.558, G=0.442; (AA=14, AG/GG=29) |
| rs1800497 | 11 | 113 400 106 | *ANKK1* | G=0.806, A=0.194 | e.g., Hayden et al. 2010 | G=0.767, A=0.233; (GG=27, GA/AA=16) |

AS1: antisense RNA to *ADORA2A*; UTR: untranslated region; for used databases see following links: UCSC Genome Browser (GRCh38/hg38): <http://genome-euro.ucsc.edu/>; dbSNP: <https://www.ncbi.nlm.nih.gov/snp/>; gnomAD (The Genome Aggregation Database; Europe): <http://gnomad.broadinstitute.org/>; PubMed: <https://www.ncbi.nlm.nih.gov/pubmed/>; 1000genomes (Europe): <http://www.1000genomes.org/>; **PubMed Reference list:** Bachmann V, Klaus F, Bodenmann S, Schäfer N, Brugger P, Huber S, et al. (2012) Cerebr Cortex 22(4):962–70; Betcheva ET, Mushiroda T, Takahashi A, Kubo M, Karachanak SK, Zaharieva IT, et al. (2009) J Hum Genet 54(2):98-107; Blasi G, Lo Bianco L, Taurisano P, Gelao B, Romano R, Fazio L, et al. (2009) J Neurosci 29(47):14812-9; Campbell NG, Zhu C-B, Lindler KM, Yaspan BL, Kistner-Griffin E, NIH ARRA Consortium, et al. (2013) Bioinformatics 21(13):2933–42; Clarke TK, Weiss AR, Ferarro TN, Kampman KM, Dackis CA, Pettinati HM, et al. (2014) Ann Hum Genet 78(1):33-9; Dutra GP, Ottoni GL, Lara DR, Bogo MR (2010;) Rev Brasil Psiquiatria 32(3):275–8; Figler RA, Wang G, Srinivasan S, Jung DY, Zhang Z, Pankow JS, et al. (2011) Diabetes 60(2):669–79; Ghosh J, Pradhan S, Mittal B (2013) Neuromolecular Med 15, 61–73; Hayden EP, Klein DN, Dougherty LR, Olino TM, Laptook RS, Dyson MW, et al. (2010) Psychiatr Genet 20(6):304-10; Hettinger JA, Liu X, Holden JJ (2008) J Autism Development Disorders 38(1):14-9; Hirvonen M, Laakso A, Någren K, Rinne JO, Pohjalainen T, Hietala J (2004) Mol Psychiatry 9(12):1060–1; Hohoff C, Garibotto V, Elmenhorst D, Baffa A, Kroll T, Hoffmann A, et al. (2014) Neuropsychopharmacology 39(13):2989–99; Kim SH, Kim YK, Park HW, Kim SH, Kim SH, Ye YM, et al. (2009) Resp Medicine 103(3):356–63; Kim SH, Nam EJ, Kim YK, Ye YM, Park HS (2010) British J Dermatology 163(5):977–85; Sipilä T, Kananen L, Greco D, Donner J, Silander K, Terwilliger JD, et al. (2010) Biol Psychiatry. 2010 Jun 15;67(12):1163-70; Todt U, Netzer C, Toliat M, Heinze A, Goebel I, Nürnberg P, et al. (2007) Hum Genet 125(3):265-79; Whitmer AJ, Gotlib IH (2012) Cogn Affect Behav Neurosci 12:741-747; Zhang Y, Bertolino A, Fazio L, Blasi G, Rampino A, Romano R, et al. (2007) Proc Natl Acad Sci U S A 104(51):20552-7.

**Table S2:** Genotyping details for variants of adenosinergic/dopaminergic genes *ADORA2B*, *ADORA3*, *ADA*, and *DRD2* with primer sequences, RFLP assay and sequencing conditions

| Gene and variation | Primer sequence (5' to 3' direction)  (F = Forward; R = Reverse) | Amplicon length | Annealing Temp. (T) | Polymerase  type | Restriction  enzyme | Digest  T | Fragment lengths  after restriction digest | Primer used for sequencing (at T) |
| --- | --- | --- | --- | --- | --- | --- | --- | --- |
| Adenosinergic: |  |  |  |  |  |  |  |  |
| *ADORA2B* |  |  |  |  |  |  |  |  |
| rs758857 | F: AATTCTCACTCACCACGGCC R: GCATTCATCCACAATGTGCTTT | 251 bp | 60 °C | Standard | HhaI | 37 °C | A allele: 209, 42 G allele: 159, 50, 42 | Forward (60 °C) |
| rs2535609 | F: AAAGTACAAAAAGGCCCAAAAGG R: GTCCACAGGAAAAAAATCAAAACA | 251 bp | 60 °C | HotStart | BstNI | 60 °C | A allele: 78, 62, 56, 55 G allele: 117, 78, 56 | Reverse (60 °C) |
| *ADORA3* |  |  |  |  |  |  |  |  |
| rs1890245 | F: CCATTTATATGTCAGGGAGCGG R: TTAGGCCAACTTTAGCACAAGGT | 252 bp | 64 °C | Standard | NlaIII | 37 °C | A allele: 134, 70, 48 T allele: 204, 48 | Reverse (64 °C) |
| rs35254520 | F: CCACTGCGGAGGGAATCTC R: GAGTTCTGGTTCAGCGACCC | 151 bp | 64 °C | Standard | 1. TaqI  2. AlwI | 65 °C  55 °C | Taql: C allele: 151  G allele: 102, 49  Alwl: C allele: 94, 57  G allele: 151 | Forward (64 °C) |
| rs2786995 | F: TGTGGACAGAGGCTCCAGATC R: TGCACAAAATCAGCTTGCTCA | 251 bp | 64 °C | HotStart | 1. HhaI  2. AluI | 37 °C  37 °C | Hhal: C allele: 140, 52, 32, 27  A or G allele: 140, 79, 32  AluI: A allele: 98, 59, 59, 22, 13 C or G allele: 157, 59, 22, 13 | Reverse (64 °C) |
| rs10776727 | F: TGACGCTTTGCTGAGCACA R: TGATTCCCAGACAGTCGCC | 358 bp | 64 °C | Standard | BseYI | 37 °C | A allele: 300, 31, 27 C allele: 331, 27 | Forward (64 °C) |
| rs1544224 | F: GCTAAGCTGGCAGAAAGATTGCAT R: GGTCACTTCCAGCCCCTTTATG | 401 bp | 64 °C | Standard | AciI | 37 °C | C allele: 332, 42, 27 T allele: 374, 27 | Reverse (64 °C) |
| rs2229155 | F: CTGCCTTTATCTATCATCAACTGCATC R: GCATACAGGCCCTCAAGTGTTTG | 301 bp | 64 °C | Standard | AluI | 37 °C | C allele: 247, 54 T allele: 135, 112, 54 | Forward (64 °C) |
| *ADA* |  |  |  |  |  |  |  |  |
| rs73598374 | F: GGCACCATGGCCCAGAC R: TGATTAGCCCGCAAGCAAG | 202 bp | 60 °C | HotStart | 1. TaqI  2. AgsI | 37 °C  37 °C | TaqI: A allele: 202;  G allele: 176, 26  Agsl: A allele: 175, 27  G allele: 202 | Reverse (60 °C) |
| rs427483 | F: CCCTCCCCTTTCTGGCAG R: CAAGTGGGCATCCTAGCACC | 251 bp | 64 °C | Standard | AciI | 37 °C | C allele: 230, 21 G allele: 127, 103, 21 | Forward (64 °C) |

To be continued

**Table S2:** continued

| Dopaminergic: |  |  |  |  |  |  |  |  |
| --- | --- | --- | --- | --- | --- | --- | --- | --- |
| *DRD2* |  |  |  |  |  |  |  |  |
| rs4648317 | F: AGCTGTTCTCAGGCCAGCTTTG  R: TGTCCCTGCCCTTTGCTCCT | 251 bp | 71 °C | Standard | HpyAV | 37 °C | T allele: 139, 76, 36  C allele: 175, 76 | Reverse (64 °C) |
| rs7131056 | F: ACACAGGTGGTGAGACCTGAAGCTG R:GAAAGTGAAGAAGATAAACCTAGCA  TAGGTAGAAAATATG | 115 bp | 64 °C | Standard | BsII | 37 °C | G allele: 71, 22, 22  T allele: 93, 22 | Reverse (64 °C) |
| rs4936272 | F:TCTCTGTGTTTCCAAGAATAGCCA  R: AAACAGGGAGTGAGTGAGTCACC | 294 bp | 54 °C | HotStart | HypCH4V | 37 °C | A allele: 174, 67, 53  G allele: 227, 67 | Forward (54 °C) |
| rs4245146 | F: CTTCCATCTGCCCATACCAAG  R: CTCCAGCCTCCAAAGACAGC | 281 bp | 60 °C | Standard | AciI | 37 °C | G allele: 102, 63, 58, 58  A allele: 116, 102, 63 | Reverse (54 °C) |
| rs17529477 | F: ACAGTGTGCAAGACAGTGTCCAG  R: TGGATCCCACCCTAGTTCCC | 201 bp | 64 °C | Standard | BseNI | 65 °C | C allele: 68, 61, 52, 20  T allele: 113, 68, 20 | Forward (64 °C) |
| rs55900980 | F: AGTTGGTGACGCTAAGCCCAGA  R: CACCAGTCCCCGCAGCTG | 131/130 bp | 69 °C | Standard | HgaI | 37 °C | C allele: 94, 20, 17  Deletion allele: 113, 17 | Forward (64 °C) |
| rs1076560 | F: ATTGAGGCTGCATGAGGATTG  R: CTCTGGGTAAAGCCGGACAA | 209 bp | 64 °C | HotStart | HphI | 37 °C | T allel: 122, 53, 34  G allel: 122, 87 | Forward (64 °C) |
| rs6275 | F: GAGCTGGAGATGGAGATGCT  R: GGAATGGGACCTTTCACAGA | 360 bp | 66 °C | Standard | NlaIII | 37 °C | T allele: 117, 109, 51, 47, 36  C allele: 160, 117, 47, 36 | Reverse (64 °C) |
| rs6277 | F: GAGCTGGCGATGGAGATGCT  R: CTCTGGTTTGGCAGGGCTGTC | 147 bp | 66 °C | HotStart | BcgI | 37 °C | T allele: 116, 31  C allele: 84, 32, 31 | Forward (64 °C) |
| rs1800497 | F: ACTCCATCCTCGACGTCCAGCT  R: TGACCCCAATGCTGCAGAGC | 222 bp | 64 °C | HotStart | TaqI | 65 °C | C allele: 188, 24, 10  T allele: 212, 10 | Reverse (64 °C) |

RFLP: restriction fragment length polymorphism; underscoring in primer sequences indicate mutated bases to create restriction sites

**Table S3.** Pairwise linkage disequilibrium (LD) analysis of *ADORA1*, *ADORA2A*, *ADORA2B*, *ADORA3*, *ADA*, and *DRD2* variants

| Adenosinergic system variants: | | LD statistics: | |  | Dopaminergic system variants: | | LD statistics: | |
| --- | --- | --- | --- | --- | --- | --- | --- | --- |
| Variant 1 | Variant 2 | D' | r^2 |  | Variant 1 | Variant 2 | D' | r^2 |
| A2A_rs5751862 | A2A_rs5760405 | 1 | 0.224 |  | D2_rs1800497 | D2_rs6277 | 0.656 | 0.165 |
| A2A_rs5751862 | A2A_rs2236624 | 0.782 | 0.243 |  | D2_rs1800497 | D2_rs6275 | 1 | 0.098 |
| A2A_rs5751862 | A2A_rs5751876 | 0.889 | 0.656 |  | D2_rs1800497 | D2_rs1076560* | 1 | 0.813* |
| A2A_rs5751862 | A2A_rs4822492 | 0.889 | 0.656 |  | D2_rs1800497 | D2_rs55900980* | 1 | 0.813* |
| A2A_rs5760405 | A2A_rs2236624 | 1 | 0.089 |  | D2_rs1800497 | D2_rs17529477 | 0.035 | 0 |
| A2A_rs5760405 | A2A_rs5751876 | 1 | 0.186 |  | D2_rs1800497 | D2_rs4245146 | 0.2 | 0.008 |
| A2A_rs5760405 | A2A_rs4822492 | 1 | 0.186 |  | D2_rs1800497 | D2_rs4936272 | 0.099 | 0.002 |
| A2A_rs2236624 | A2A_rs5751876 | 1 | 0.479 |  | D2_rs1800497 | D2_rs7131056 | 0.168 | 0.009 |
| A2A_rs2236624 | A2A_rs4822492 | 1 | 0.479 |  | D2_rs1800497 | D2_rs4648317 | 0.323 | 0.007 |
| A2A_rs5751876 | A2A_rs4822492* | 1 | 1* |  | D2_rs6277 | D2_rs6275 | 1 | 0.408 |
| A1_rs1874142 | A1_rs10920568 | 1 | 0.27 |  | D2_rs6277 | D2_rs1076560 | 1 | 0.311 |
| A1_rs1874142 | A1_rs12135643 | 1 | 0.177 |  | D2_rs6277 | D2_rs55900980 | 1 | 0.311 |
| A1_rs1874142 | A1_rs3766566 | 1 | 0.201 |  | D2_rs6277 | D2_rs17529477 | 0.398 | 0.043 |
| A1_rs1874142 | A1_rs17511192 | 0.289 | 0.055 |  | D2_rs6277 | D2_rs4245146 | 0.163 | 0.013 |
| A1_rs1874142 | A1_rs6677137 | 0.17 | 0.021 |  | D2_rs6277 | D2_rs4936272 | 0.134 | 0.009 |
| A1_rs1874142 | A1_rs3753472 | 0.064 | 0.003 |  | D2_rs6277 | D2_rs7131056 | 0.034 | 0.001 |
| A1_rs10920568 | A1_rs12135643 | 1 | 0.123 |  | D2_rs6277 | D2_rs4648317 | 0.173 | 0.008 |
| A1_rs10920568 | A1_rs3766566 | 1 | 0.14 |  | D2_rs6275 | D2_rs1076560 | 1 | 0.08 |
| A1_rs10920568 | A1_rs17511192 | 0.667 | 0.184 |  | D2_rs6275 | D2_rs55900980 | 1 | 0.08 |
| A1_rs10920568 | A1_rs6677137 | 0.163 | 0.025 |  | D2_rs6275 | D2_rs17529477 | 0.643 | 0.046 |
| A1_rs10920568 | A1_rs3753472 | 0.205 | 0.04 |  | D2_rs6275 | D2_rs4245146 | 0.131 | 0.003 |
| A1_rs12135643 | A1_rs3766566* | 1 | 0.878* |  | D2_rs6275 | D2_rs4936272 | 0.182 | 0.007 |
| A1_rs12135643 | A1_rs17511192 | 0.699 | 0.145 |  | D2_rs6275 | D2_rs7131056 | 0.135 | 0.005 |
| A1_rs12135643 | A1_rs6677137 | 0.599 | 0.047 |  | D2_rs6275 | D2_rs4648317 | 0.309 | 0.062 |
| A1_rs12135643 | A1_rs3753472 | 1 | 0.116 |  | D2_rs1076560* | D2_rs55900980* | 1 | 1* |
| A1_rs3766566 | A1_rs17511192 | 0.582 | 0.115 |  | D2_rs1076560 | D2_rs17529477 | 0.133 | 0.002 |
| A1_rs3766566 | A1_rs6677137 | 0.636 | 0.06 |  | D2_rs1076560 | D2_rs4245146 | 0.22 | 0.007 |
| A1_rs3766566 | A1_rs3753472 | 1 | 0.132 |  | D2_rs1076560 | D2_rs4936272 | 0.072 | 0.001 |
| A1_rs17511192 | A1_rs6677137 | 1 | 0.437 |  | D2_rs1076560 | D2_rs7131056 | 0.311 | 0.026 |
| A1_rs17511192 | A1_rs3753472 | 0.151 | 0.009 |  | D2_rs1076560 | D2_rs4648317 | 0.582 | 0.018 |
| A1_rs6677137 | A1_rs3753472 | 0.434 | 0.169 |  | D2_rs55900980 | D2_rs17529477 | 0.133 | 0.002 |
| A2B_rs758857 | A2B_rs2535609 | 1 | 0.052 |  | D2_rs55900980 | D2_rs4245146 | 0.22 | 0.007 |
| A3_rs2229155 | A3_rs1544224 | 1 | 0.706 |  | D2_rs55900980 | D2_rs4936272 | 0.072 | 0.001 |
| A3_rs2229155 | A3_rs10776727 | 1 | 0.355 |  | D2_rs55900980 | D2_rs7131056 | 0.311 | 0.026 |
| A3_rs2229155 | A3_rs2786995 | 0.135 | 0.007 |  | D2_rs55900980 | D2_rs4648317 | 0.582 | 0.018 |
| A3_rs2229155 | A3_rs35254520 | 0.134 | 0.008 |  | D2_rs17529477 | D2_rs4245146 | 0.914 | 0.461 |
| A3_rs2229155 | A3_rs1890245 | 1 | 0.156 |  | D2_rs17529477 | D2_rs4936272 | 0.912 | 0.437 |
| A3_rs1544224 | A3_rs10776727 | 1 | 0.502 |  | D2_rs17529477 | D2_rs7131056 | 0.866 | 0.235 |
| A3_rs1544224 | A3_rs2786995 | 0 | 0 |  | D2_rs17529477 | D2_rs4648317 | 1 | 0.073 |
| A3_rs1544224 | A3_rs35254520 | 0.06 | 0.001 |  | D2_rs4245146 | D2_rs4936272* | 1 | 0.952* |
| A3_rs1544224 | A3_rs1890245 | 1 | 0.221 |  | D2_rs4245146 | D2_rs7131056 | 0.573 | 0.186 |
| A3_rs10776727 | A3_rs2786995 | 0.251 | 0.06 |  | D2_rs4245146 | D2_rs4648317 | 1 | 0.132 |
| A3_rs10776727 | A3_rs35254520 | 0.434 | 0.123 |  | D2_rs4936272 | D2_rs7131056 | 0.511 | 0.156 |
| A3_rs10776727 | A3_rs1890245 | 1 | 0.44 |  | D2_rs4936272 | D2_rs4648317 | 1 | 0.138 |
| A3_rs2786995 | A3_rs35254520 | 0.933 | 0.638 |  | D2_rs7131056 | D2_rs4648317 | 1 | 0.192 |
| A3_rs2786995 | A3_rs1890245 | 0.702 | 0.232 |  |  |  |  |  |
| A3_rs35254520 | A3_rs1890245 | 0.858 | 0.494 |  |  |  |  |  |
| ADA_rs427483 | ADA_rs73598374 | 1 | 0.014 |  |  |  |  |  |

Pairwise LD was assessed by Haploview v.4.1 (Barrett et al. 2005); implemented tagging analysis revealed redundant variants. Several of these also complied with the criterion of nearly/complete LD with D’=1.0 and r^2>0.8 and thus were excluded from subsequent analyses: A2A_rs4822492, A1_rs3766566, D2_rs1076560, D2_rs55900980, D2_rs4936272 (marked by asterisks).

**Table S4.** Modulatory impact of sleep duration on *ADORA2A* rs5751876 dependent A_1_AR availability in pilot sample (N=29)*

|  | Sleep category “less sleep than mean” | | |  | Sleep category “more sleep than mean” | | |
| --- | --- | --- | --- | --- | --- | --- | --- |
|  | *ADORA2A* rs5751876 genotypes (N): | | |  | *ADORA2A* rs5751876 genotypes (N): | | |
| Human brain region: | CC (N=5) | CT (N=8) | TT (N=1) |  | CC (N=4) | CT (N=7) | TT (N=2) |
| Orbitofrontal cortex | 0.613 **↓** | 0.635 | 0.688 **↑** |  | 0.591 **↓** | 0.670 | 0.812 **↑** |
| Middle/inferior frontal gyrus | 0.702 **↓** | 0.735 | 0.892 **↑** |  | 0.704 **↓** | 0.812 | 0.896 **↑** |
| Anterior cingulate gyrus | 0.582 **↓** | 0.590 | 0.752 **↑** |  | 0.537 **↓** | 0.667 | 0.700 **↑** |
| Thalamus | 0.878 | 0.847 **↓** | 1.089 **↑** |  | 0.741 **↓** | 0.957 | 1.014 **↑** |
| Insula | 0.632 **↓** | 0.680 | 0.800 **↑** |  | 0.673 **↓** | 0.764 | 0.800 **↑** |
| Caudate | 0.681 | 0.618 **↓** | 0.796 **↑** |  | 0.525 **↓** | 0.771 | 0.871 **↑** |
| Putamen | 0.957 | 0.944 **↓** | 1.015 **↑** |  | 0.973 **↓** | 1.062 **↑** | 1.028 |
| superior temporal gyrus | 0.761 **↓** | 0.787 | 0.917 **↑** |  | 0.722 **↓** | 0.837 | 0.921 **↑** |
| Parietal cortex | 0.689 | 0.675 **↓** | 0.903 **↑** |  | 0.676 **↓** | 0.810 | 0.833 **↑** |
| Middle/inferior temporal gyrus | 0.795 **↓** | 0.849 | 0.974 **↑** |  | 0.764 **↓** | 0.851 | 0.947 **↑** |
| Superior frontal gyrus | 0.546 **↓** | 0.586 | 0.798 **↑** |  | 0.597 **↓** | 0.727 | 0.800 **↑** |
| Occipital cortex | 0.818 | 0.806 **↓** | 0.925 **↑** |  | 0.816 **↓** | 0.923 | 0.983 **↑** |
| Sensorimotor cortex | 0.521 | 0.501 **↓** | 0.721 **↑** |  | 0.537 **↓** | 0.679 | 0.730 **↑** |
| Posterior cingulate gyrus | 0.813 **↓** | 0.863 | 0.987 **↑** |  | 0.902 **↓** | 0.964 **↑** | 0.944 |
| Dorsolateral prefrontal cortex | 0.621 **↓** | 0.646 | 0.907 **↑** |  | 0.634 **↓** | 0.759 | 0.836 **↑** |
| Ventrolateral prefrontal cortex | 0.732 **↓** | 0.767 | 0.893 **↑** |  | 0.726 **↓** | 0.836 | 0.931 **↑** |
| Entorhinal cortex | 0.573 **↓** | 0.608 | 0.757 **↑** |  | 0.405 **↓** | 0.633 | 0.795 **↑** |
| Hippocampus | 0.541 | 0.484 **↓** | 0.597 **↑** |  | 0.371 **↓** | 0.582 | 0.639 **↑** |
| Amygdala | 0.539 **↓** | 0.581 | 0.724 **↑** |  | 0.542 **↓** | 0.617 | 0.666 **↑** |

*Pilot sample as described in Hohoff et al. 2014; grey tonality and arrows display A_1_AR availabilities (*BP*_ND_) per brain region: dark grey and ↑ = highest value; middle grey = middle value; lightest grey and ↓ = lowest value.

**Table S5.** Modulatory impact of sleep duration on *ADORA2A* rs5751876 dependent A_1_AR availability in present sample (N=43)

|  | Sleep category “less sleep than mean” | | |  | Sleep category “more sleep than mean” | | |
| --- | --- | --- | --- | --- | --- | --- | --- |
|  | *ADORA2A* rs5751876 genotypes (N): | | |  | *ADORA2A* rs5751876 genotypes (N): | | |
| Human brain region: | CC (N=6) | CT (N=9) | TT (N=6) |  | CC (N=5) | CT (N=13) | TT (N=4) |
| Precentral gyrus | 0.723 **↑** | 0.557 **↓** | 0.635 |  | 0.627 | 0.612 **↓** | 0.768 **↑** |
| Rolandic operculum | 0.863 **↑** | 0.588 **↓** | 0.704 |  | 0.717 | 0.692 **↓** | 0.816 **↑** |
| Supplementary motor area | 0.705 **↑** | 0.563 **↓** | 0.624 |  | 0.604 | 0.591 **↓** | 0.719 **↑** |
| Olfactory cortex | 0.502 **↑** | 0.309 **↓** | 0.435 |  | 0.416 | 0.375 **↓** | 0.457 **↑** |
| Superior frontal gyrus | 0.818 **↑** | 0.597 **↓** | 0.679 |  | 0.653 | 0.626 **↓** | 0.776 **↑** |
| Middle frontal gyrus | 0.906 **↑** | 0.680 **↓** | 0.760 |  | 0.764 | 0.736 **↓** | 0.895 **↑** |
| Inferior frontal gyrus | 0.870 **↑** | 0.642 **↓** | 0.731 |  | 0.743 | 0.732 **↓** | 0.904 **↑** |
| **Gyrus rectus** | 0.752 **↑** | 0.526 **↓** | 0.578 |  | **0.551 ↓** | **0.561** | **0.687 ↑** |
| Insula | 0.781 **↑** | 0.584 **↓** | 0.636 |  | 0.663 | 0.655 **↓** | 0.774 **↑** |
| **Anterior cingulate cortex** | 0.602 **↑** | 0.443 **↓** | 0.540 |  | **0.463 ↓** | **0.499** | **0.581 ↑** |
| **Middle cingulate cortex** | 0.726 **↑** | 0.515 **↓** | 0.604 |  | **0.554 ↓** | **0.609** | **0.701 ↑** |
| Posterior cingulate cortex | 0.817 **↑** | 0.546 **↓** | 0.689 |  | 0.691 | 0.713 **↑** | 0.685 **↓** |
| **Hippocampus-parahippoc.** | 0.629 **↑** | 0.395 **↓** | 0.522 |  | **0.466 ↓** | **0.499** | **0.531 ↑** |
| **Amygdala** | 0.631 **↑** | 0.410 **↓** | 0.494 |  | **0.408 ↓** | **0.454** | **0.509 ↑** |
| **Calcarine fissures** | 0.948 **↑** | 0.728 **↓** | 0.861 |  | **0.794 ↓** | **0.812** | **0.953 ↑** |
| Cuneus | 0.996 **↑** | 0.748 **↓** | 0.890 |  | 0.873 | 0.854 **↓** | 0.990 **↑** |
| **Lingual gyrus** | 0.909 **↑** | 0.668 **↓** | 0.828 |  | **0.741 ↓** | **0.770** | **0.852 ↑** |
| **Occipital lobe** | 0.927 **↑** | 0.685 **↓** | 0.821 |  | **0.775 ↓** | **0.795** | **0.898 ↑** |
| **Fusiform gyrus** | 0.824 **↑** | 0.613 **↓** | 0.748 |  | **0.698 ↓** | **0.707** | **0.801 ↑** |
| Postcentral gyrus | 0.669 **↑** | 0.469 **↓** | 0.551 |  | 0.587 | 0.538 **↓** | 0.721 **↑** |
| Supramarginal gyrus | 0.955 **↑** | 0.674 **↓** | 0.757 |  | 0.841 | 0.786 **↓** | 0.902 **↑** |
| Angular gyrus | 1.032 **↑** | 0.751 **↓** | 0.831 |  | 0.905 | 0.867 **↓** | 1.018 **↑** |
| Precuneus | 0.894 **↑** | 0.650 **↓** | 0.767 |  | 0.785 | 0.769 **↓** | 0.932 **↑** |
| **Paracentral lobule** | 0.617 **↑** | 0.425 **↓** | 0.504 |  | **0.522 ↓** | **0.527** | **0.572 ↑** |
| Caudate | 0.616 **↑** | 0.450 **↓** | 0.548 |  | 0.531 | 0.538 | 0.521 **↓** |
| Putamen | 1.072 **↑** | 0.853 **↓** | 0.970 |  | 0.920 | 0.881 **↓** | 1.108 **↑** |
| **Pallidum** | 0.761 **↑** | 0.527 **↓** | 0.706 |  | **0.580 ↓** | **0.667** | **0.723 ↑** |
| **Thalamus** | 0.989 **↑** | 0.747 **↓** | 0.752 |  | **0.779 ↓** | **0.808** | **0.907 ↑** |
| Heschl gyrus | 0.847 **↑** | 0.603 **↓** | 0.643 |  | 0.706 | 0.657 **↓** | 0.866 **↑** |
| Parietal | 0.848 **↑** | 0.598 **↓** | 0.693 |  | 0.727 | 0.701 **↓** | 0.888 **↑** |
| **Temporal lobe** | 0.966 **↑** | 0.700 **↓** | 0.804 |  | **0.798 ↓** | **0.799** | **0.913 ↑** |

Bold face indicates brain regions with gene-dose like distribution of rs5751876 genotype dependent A_1_AR availability; grey tonality and arrows display A_1_AR availabilities (*BP*_ND_) per brain region: dark grey and ↑ = highest value; middle grey = middle value; lightest grey and ↓ = lowest value.

**Table S6.** Distribution of *ADORA1*, *ADORA2B*, *ADORA3*, *ADA*, and *DRD2* genotype groups between samples

| Gene and variation | Present male/female sample | Pilot male sample (Hohoff et al. 2014) | P-value |
| --- | --- | --- | --- |
| Adenosinergic: |  |  |  |
| *ADORA1* |  |  |  |
| rs1874142 | GG=16, GAAA=27 | GG=8, GAAA=20 | n.s. |
| rs10920568 (=rs2228079) | TT=20, TGGG=23 | TT=19, TGGG=9 | n.s. |
| rs12135643 | CC=26, CAAA=17 | CC=18, CAAA=10 | n.s. |
| rs17511192 | CC=11, CTTT=32 | CC=7, CTTT=21 | n.s. |
| rs6677137 | TT=19, TCCC=24 | TT=11, TCCC=17 | n.s. |
| rs3753472 | TT=21, TCCC=22 | TT=12, TCCC=16 | n.s. |
| *ADORA2B*: |  |  |  |
| rs758857 | AA=27, AGGG=16 | AA=19, AGGG=9 | n.s. |
| rs2535609 | AA=29, AGGG=14 | AA=16, AGGG=12 | n.s. |
| *ADORA3*: |  |  |  |
| rs1890245 | AA=19, ATTT=24 | AA=16, ATTT=12 | n.s. |
| rs35254520 | CC=13, CGGG=30 | CC=14, CGGG=14 | n.s. |
| rs2786995 | CC=9, CGGG=30 | CC=7, CGGG=20 | n.s. |
| rs10776727 | AA=9, ACCC=34 | AA=7, ACCC=21 | n.s. |
| rs1544224 | GG=22, GAAA=21 | GG=16, GAAA=12 | n.s. |
| rs2229155 | GG=24, GAAA=19 | GG=18, GAAA=10 | n.s. |
| *ADA*: |  |  |  |
| rs73598374 | GG=38, GAAA=5 | GG=24, GAAA=4 | n.s. |
| rs427483 | CC=29, CGGG=14 | CC=17, CGGG=11 | n.s. |
| Dopaminergic: |  |  |  |
| *DRD2*: |  |  |  |
| rs4648317 | GG=30, GAAA=13 | GG=19, GAAA=9 | n.s. |
| rs7131056 | CC=12, CAAA=31 | CC=13, CAAA=15 | n.s. |
| **rs4245146** | **CC=15, CTTT=28** | **CC=1, CTTT=27** | **0.002** |
| rs17529477 | GG=24, GAAA=19 | GG=10, GAAA=18 | n.s. |
| rs6275 | GG=26, GAAA=17 | GG=16, GAAA=12 | n.s. |
| rs6277 | AA=14, AGGG=29 | AA=12, AGGG=16 | n.s. |
| **rs1800497** | **GG=27, GAAA=16** | **GG=24, GAAA=4** | **0.036** |

Statistics: distribution of *ADORA1*, *ADORA2B*, *ADORA3*, *ADA*, and *DRD2* genotype groups were compared in present vs. pilot sample by nonparametric statistics (Fisher’s exact test); n.s.: not significant (P>0.05); bold indicates significant results.

**Table S7.** Exploratory analysis of *DRD2* rs4245146 modulation of *ADORA2A* rs5751876 dependent A_1_AR availability in present sample

|  | *ADORA2A* rs5751876 CC-carrier (N=10): | | | *ADORA2A* rs5751876 CT-/TT-carrier (N=24): | | |
| --- | --- | --- | --- | --- | --- | --- |
|  | Mean A_1_AR availability in | |  | Mean A_1_AR availability in | |  |
| *DRD2* rs4245146: | CCs (N=4) | CT/TTs (N=7) |  | CCs (N=11) | CT/TTs (N=21) |  |
| Human brain region: |  |  | P-Values |  |  | P-Values |
| Precentral gyrus | 0.817 **↑** | 0.601 | **0.020*** | 0.570 **↓** | 0.646 | 0.223 |
| Rolandic operculum | 0.910 **↑** | 0.732 | **0.090** | 0.629 **↓** | 0.708 | 0.236 |
| Supplementary motor area | 0.776 **↑** | 0.592 | **0.040*** | 0.553 **↓** | 0.633 | 0.161 |
| Olfactory cortex | 0.510 **↑** | 0.436 | 0.788 | 0.364 **↓** | 0.385 | 0.718 |
| Superior frontal gyrus | 0.869 **↑** | 0.671 | **0.018*** | 0.588 **↓** | 0.677 | 0.289 |
| Middle frontal gyrus | 0.961 **↑** | 0.773 | **0.029*** | 0.688 **↓** | 0.775 | 0.193 |
| Inferior frontal gyrus | 0.916 **↑** | 0.753 | 0.147 | 0.659 **↓** | 0.764 | 0.106 |
| Gyrus rectus | 0.755 **↑** | 0.607 | 0.160 | 0.541 **↓** | 0.585 | 0.527 |
| Insula | 0.820 **↑** | 0.674 | 0.111 | 0.587 **↓** | 0.677 | 0.144 |
| Anterior cingulate cortex | 0.627 **↑** | 0.488 | **0.089** | 0.446 **↓** | 0.530 | 0.114 |
| Middle cingulate cortex | 0.735 **↑** | 0.598 | **0.061** | 0.541 **↓** | 0.620 | 0.184 |
| Posterior cingulate cortex | 0.852 **↑** | 0.707 | **0.083** | 0.594 **↓** | 0.692 | 0.235 |
| Hippocampus-parahippoc. | 0.616 **↑** | 0.520 | 0.270 | 0.425 **↓** | 0.506 | 0.143 |
| Amygdala | 0.639 **↑** | 0.467 | **0.090** | 0.380 **↓** | 0.496 | **0.048*** |
| Calcarine fissures | 1.015 **↑** | 0.800 | **0.072** | 0.766 **↓** | 0.841 | 0.367 |
| Cuneus | 1.070 **↑** | 0.866 | **0.078** | 0.783 **↓** | 0.882 | 0.177 |
| Lingual gyrus | 0.958 **↑** | 0.761 | **0.041*** | 0.699 **↓** | 0.796 | 0.203 |
| Occipital lobe | 0.977 **↑** | 0.790 | **0.034*** | 0.733 **↓** | 0.807 | 0.251 |
| Fusiform gyrus | 0.844 **↑** | 0.722 | **0.081** | 0.633 **↓** | 0.735 | **0.083** |
| Postcentral gyrus | 0.747 **↑** | 0.566 | **0.015*** | 0.485 **↓** | 0.575 | 0.133 |
| Supramarginal gyrus | 1.008 **↑** | 0.844 | **0.057** | 0.697 **↓** | 0.798 | 0.152 |
| Angular gyrus | 1.092 **↑** | 0.907 | **0.030*** | 0.784 **↓** | 0.880 | 0.187 |
| Precuneus | 0.956 **↑** | 0.781 | 0.157 | 0.701 **↓** | 0.785 | 0.237 |
| Paracentral lobule | 0.700 **↑** | 0.502 | **0.053** | 0.455 **↓** | 0.523 | 0.227 |
| Caudate | 0.639 **↑** | 0.542 | 0.340 | 0.465 **↓** | 0.538 | 0.287 |
| Putamen | 1.068 **↑** | 0.966 | 0.348 | 0.844 **↓** | 0.957 | 0.121 |
| Pallidum | 0.681 **↑** | 0.677 | 0.983 | 0.570 **↓** | 0.680 | 0.165 |
| Thalamus | 1.010 **↑** | 0.827 | 0.113 | 0.762 **↓** | 0.809 | 0.534 |
| Heschl gyrus | 0.934 **↑** | 0.696 | **0.007*** | 0.623 **↓** | 0.688 | 0.333 |
| Parietal | 0.935 **↑** | 0.712 | **0.018*** | 0.643 **↓** | 0.721 | 0.293 |
| Temporal lobe | 1.006 **↑** | 0.824 | **0.034*** | 0.723 **↓** | 0.819 | 0.118 |

Bold face indicates trend and asterisked bold face significant P-values; arrows illustrate direction of *DRD2* rs4245146 genotype effect on *ADORA2A* rs5751876 dependent A_1_AR availability (↑: upregulation; ↓: downregulation). Statistics: mean A_1_AR availability in *DRD2* rs4245146 genotype groups CC vs. CT+TT was compared by parametric/nonparametric statistics (Student’s T test/ Mann-Whitney U-test) as appropriate.

**
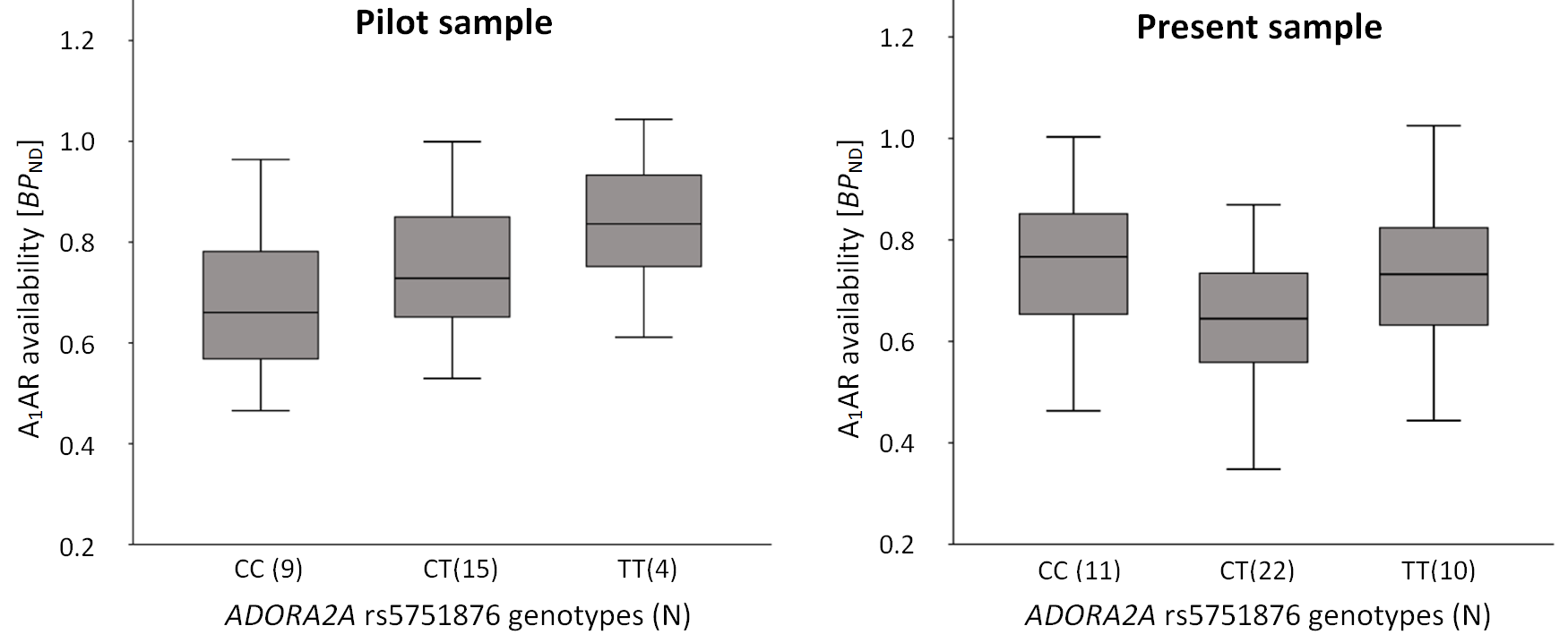
**

**Suppl. Figure S1:** Comparison of the *ADORA2A* rs5751876 genotype effect on brain A_1_AR availability data in the present sample (N=43) and the pilot sample (N=28; Hohoff et al. 2014). Consistent CT to TT (lower to higher A_1_AR availability) gene-dose effects were detected in both samples, whereas the CCs scored high in the present but low in the pilot sample. Together these results formed a U-shaped curve in the present sample in contrast to a gene-dose curve in the pilot sample. Box plots represent mean A_1_AR availability data calculated from all 31 brain regions (present sample) or all 19 brain regions (pilot sample).


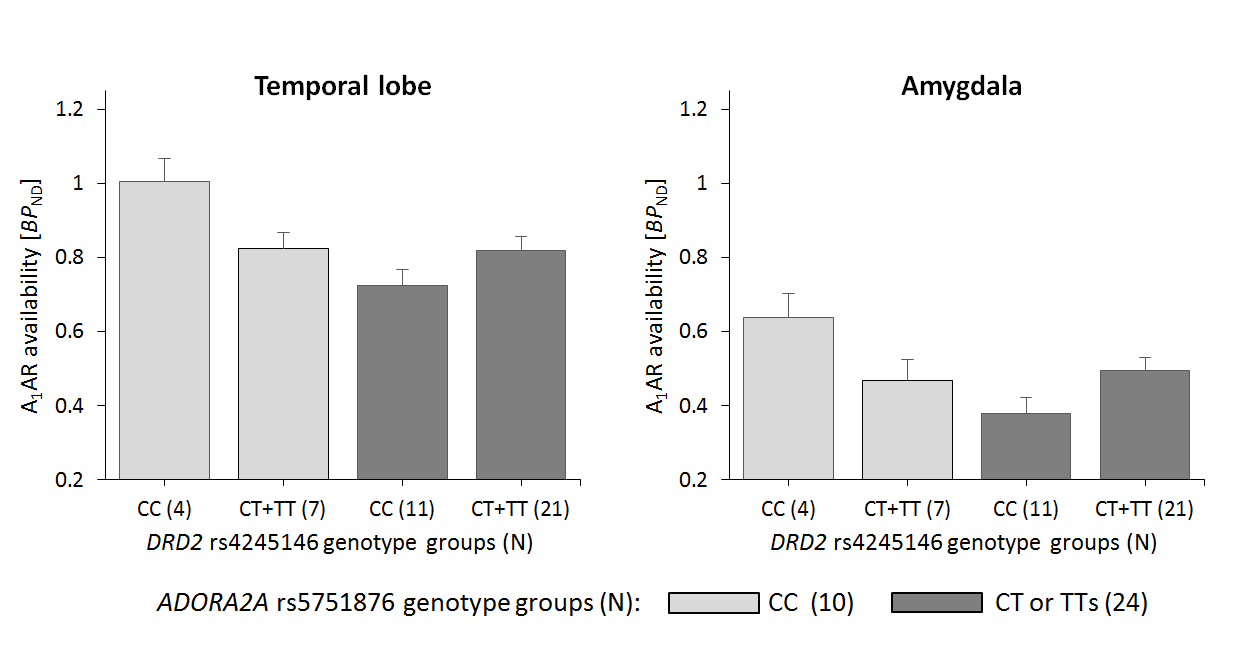


**Suppl. Figure S2:** Modulatory impact of *DRD2* variant rs4245146 on *ADORA2A* rs5751876 dependent A_1_AR availability presented as bar plots (based on Table S5) exemplary for anxiety-related brain regions temporal lobe (rs4245146 x rs5751876: P=0.016) and amygdala (rs4245146 x rs5751876: P=0.012). Statistics: exploratory (uncorrected) univariate analysis with brain region specific mean A_1_AR availability as dependent variable and genotype groups as independent factors and interaction term.
